# Integrating physicomechanical and biological strategies for BTE: biomaterials-induced osteogenic differentiation of MSCs

**DOI:** 10.7150/thno.84759

**Published:** 2023-05-21

**Authors:** Huixin Shi, Kaixuan Zhou, Mingfeng Wang, Ning Wang, Yiping Song, Wei Xiong, Shu Guo, Zhe Yi, Qiang Wang, Shude Yang

**Affiliations:** 1 Department of Plastic Surgery, The First Hospital of China Medical University, Shenyang 110001, China.; 2 Liaoning Provincial Key Laboratory of Oral Diseases, School and Hospital of Stomatology, China Medical University, Shenyang 110001, China.; 3 Department of Plastic Surgery, The First Affiliated Hospital of Medical College of Shihezi University, Shihezi, Xinjiang 832008, China.

**Keywords:** osteogenesis, physicomechanical stimuli, biomaterials, mesenchymal stem cells, mechanisms

## Abstract

Large bone defects are a major global health concern. Bone tissue engineering (BTE) is the most promising alternative to avoid the drawbacks of autograft and allograft bone. Nevertheless, how to precisely control stem cell osteogenic differentiation has been a long-standing puzzle. Compared with biochemical cues, physicomechanical stimuli have been widely studied for their biosafety and stability. The mechanical properties of various biomaterials (polymers, bioceramics, metal and alloys) become the main source of physicomechanical stimuli. By altering the stiffness, viscoelasticity, and topography of materials, mechanical stimuli with different strengths transmit into precise signals that mediate osteogenic differentiation. In addition, externally mechanical forces also play a critical role in promoting osteogenesis, such as compression stress, tensile stress, fluid shear stress and vibration, etc. When exposed to mechanical forces, mesenchymal stem cells (MSCs) differentiate into osteogenic lineages by sensing mechanical stimuli through mechanical sensors, including integrin and focal adhesions (FAs), cytoskeleton, primary cilium, ions channels, gap junction, and activating osteogenic-related mechanotransduction pathways, such as yes associated proteins (YAP)/TAZ, MAPK, Rho-GTPases, Wnt/β-catenin, TGFβ superfamily, Notch signaling. This review summarizes various biomaterials that transmit mechanical signals, physicomechanical stimuli that directly regulate MSCs differentiation, and the mechanical transduction mechanisms of MSCs. This review provides a deep and broad understanding of mechanical transduction mechanisms and discusses the challenges that remained in clinical translocation as well as the outlook for the future improvements.

## 1. Introduction

Bones have remarkable healing potential and are able to regenerate themselves upon injury or defect. Small bone defects achieve self-healing with the formation of new bone. However, large bone defects caused by trauma, tumor or infection, such as osteoporosis and osteonecrosis, are far beyond their self-healing capability, thereby requiring grafts to promote defect repair and bone regeneration 1, 2. Although autologous bone transplantation is considered to be an optimal strategy for treating bone defects, its clinical application is limited by the insufficiency of autologous bone transplantation and the morbidity of the donor site 3. Bone allografts have a high risk of immune rejection and are also abandoned 3. Thus, tissue engineered bone seems to be a promising alternative 3, 4.

In recent years, BTE based on MSCs has aroused much interest 4. These cells are not only easy to obtain, but also have the potential to differentiate into lineages, including osteoblasts, chondrocytes, adipocytes, and muscle cells 3, 5. However, how to precisely control the fate of MSCs is still an important subject for investigation in BTE. The conventional approach induces stem cells to differentiate into various lineages by transmitting biochemical signaling molecules 6. Nonetheless, the biosafety of these biochemical factors still needs to be evaluated. And how to achieve temporally and spatially controlled release has not been solved 3, 7. Therefore, the regulation of MSCs osteogenic differentiation by physical and mechanical strategies is considered to be a safer and more stable approach.

**Figure 1:**
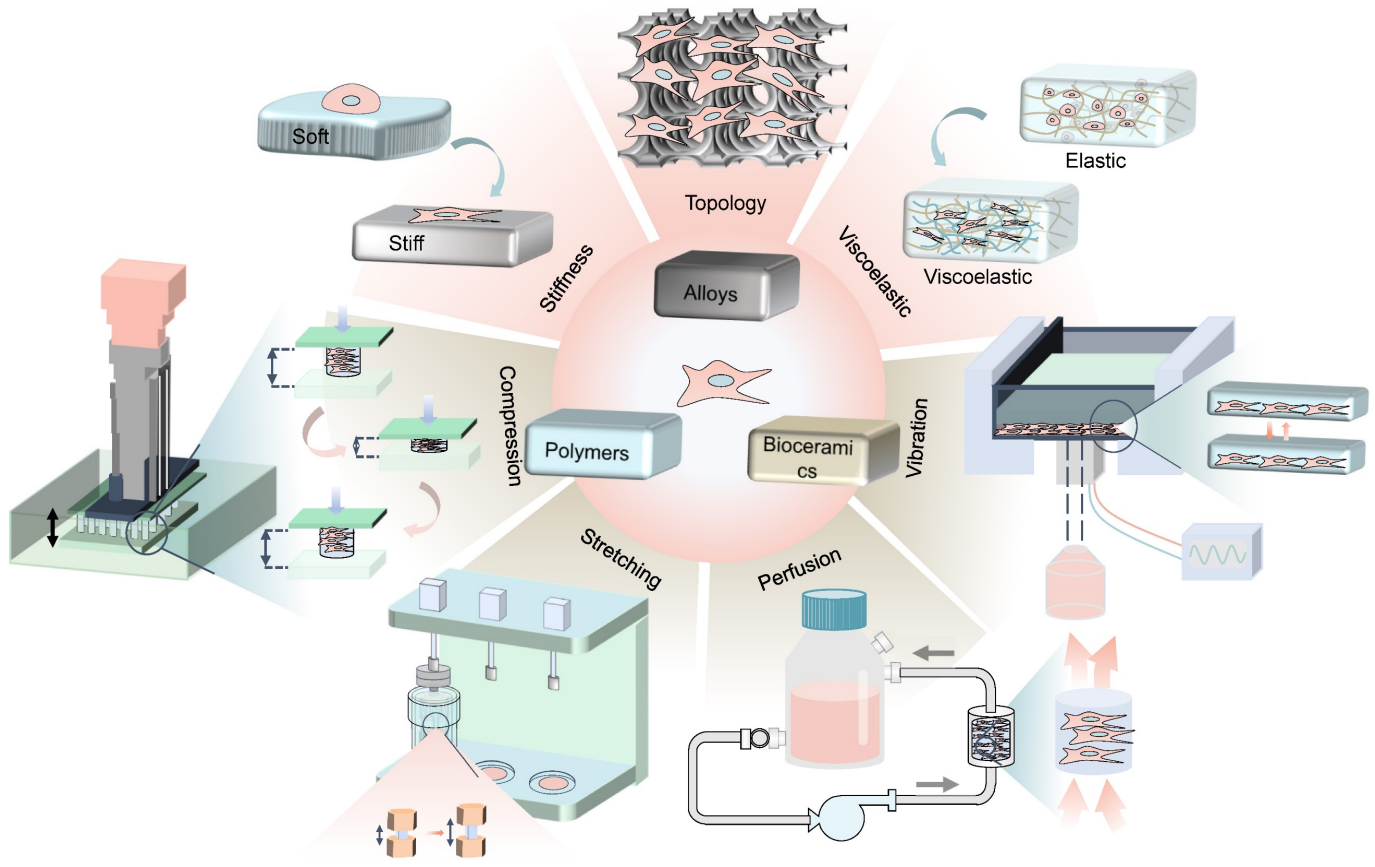
Schematic illustration of physicomechanical stimuli based on biomaterials to induce osteogenic differentiation of MSCs.

Physicomechanical stimuli is divided into internal forces generated by the cell-laden biomaterials (such as stiffness, viscoelasticity, and topography) and externally mechanical forces (such as compression stress, tensile stress, fluid shear stress and vibration) 6, which has substantial effects on stem cell differentiation through different mechanisms** (Figure 1)**. For instance, high stiffness of biomaterials drives MSCs into the osteogenic lineage, while the low stiffness promotes adipogenic differentiation 8. Various rough topographies, such as groove or ridge structures 9, have been demonstrated to promote osteogenic differentiation as well. However, static culture only allows oxygen and nutrients to slowly diffuse to the center of the scaffold, which causes some cells to undergo apoptosis due to insufficient supply of nutrients and oxygen 2. In contrast, dynamic culture with bioreactors that provide mechanical loads not only allows for more uniform cell distribution and adequate nutrition, but also has been shown to better promote osteogenic differentiation of MSCs 2. Therefore, dynamic cultivation by applying external mechanical force is also widely concerned in the field of BTE recently.

Mechanobiology is an emerging field, which integrates both physicomechanical and biological strategies, including receiving mechanical signals and transforming extracellular mechanical signals into intracellular biological ones 3. Mechanoreceptors on cell surface sense mechanical cues and subsequently transmit signals to the nucleus through dynamic regulation of cytoskeletal integrity and tension. The nucleus responds to the signals by up-regulating or down-regulating the expression of genes associated with mechanical stimulation 3, 5, 10. In this review, we first listed different biomaterials and the approaches to alter their mechanical properties, in order to dictate MSCs differentiation towards osteogenic lineage. Then, we summarized physicomechanical stimuli that drove osteogenesis, including stiffness, viscoelasticity, and topological structure of materials, as well as external mechanical forces. Subsequently, we illustrated how MSCs converted mechanical stimuli into biochemical signals, and several mechanotransduction-associated signaling pathways during osteogenesis. Finally, we discussed the major challenges that might encounter in the future transformation of MSCs-laden biomaterials based on mechanical conduction in BTE.

## 2. Biomaterials-induced physicomechanical stimuli towards MSCs

### 2.1 Internal mechanical stimulation on MSCs-laden biomaterials

Biomaterials regulate cell behavior by mimicking the natural ECM 11, and their physicomechanical properties are regarded as the major stimuli that governs fate decisions of MSCs. This section will focus on the processing methods of biomaterials for inducing osteogenesis of MSCs including hydrogels and other polymers, bioceramics, metal and alloys. Furthermore, as the primary means for directing osteogenesis, the modulation on their stiffness, viscoelasticity, and topography will be detailed below.

#### 2.1.1 Biomaterials processing method

##### 2.1.1.1 Hydrogels and other polymers

Hydrogels are widely used as matrix material in BTE and regenerative medicine, especially in three-dimensional (3D) microenvironment 12. Compared with bioceramic and metal-based materials, hydrogels have become the mainstream matrix materials for inducing osteogenic differentiation of MSCs due to their adjustable stiffness 13, ease of altering morphology 14, and unique viscoelastic properties 15-17.

As is well-known, cell proliferation, migration or differentiation can be easily modulated by changing the stiffness of the hydrogel. Cells tend to differentiate into osteogenic lineage on a stiff matrix 18, while soft matrix enhances cell proliferation and migration 19. Therefore, hydrogels play a critical role in BTE for their adjustability of stiffness 17. In recent years, the fabrication of hydrogels has been extensively explored, with most attention on how to precisely regulate the physicomechanical properties of hydrogels in a simple way. The traditional method is to adjust the proportion of each component of the hydrogel. For example, polyacrylamide (PA) hydrogels' physiological stiffness can be adjusted by controlling the ratio of acrylamide to bis-acrylamide 20. Hadden WJ et al. developed an approach of polymerization control to synthesize linear stiffness gradient PA hydrogels, which was simpler and cheaper than other synthesis methods 21. By adjusting the concentration of hydroxyapatite (HAp) in the methacrylated hyaluronic acid hydrogel, a matrix material with tunable stiffness can be formed, which alters the cell volume, differentiation and cell fate decisions 22. Furthermore, some emerging technologies have aroused increasing attention nowadays. Wet spinning method allows the fabrication of gelatin-based microstrip hydrogels with various stiffness 23. Photoresponsive hydrogels change photoswitchable stiffness in the presence of cells through rapid cytocompatible light-based chemistries 11, this allows MSCs stiffness regulation to be investigated independently without the interference of other reagents. In later studies, soft lithography has been used to precisely control the surface morphology of hydrogels, achieving linear surface roughness variation from nanometer to micrometer on a stiffness controllable matrix 14, 20. Ultrahigh strength and high stiffness of hydrogels can also be developed through a brick-mortar-like network that composed of bacterial cellulose nanofibers and alginate-Ca^2+^ 24, or forming a hybrid scaffold with 3D PCL/nano-hydroxyapatite (nHA) scaffold 25. Macro-porous recombinant elastin-like protein substrates 4 and the combination of alginate and gelatin for bioprinting 26 are promising for the optimization of stiffness as well.

Tunable viscoelasticity and stress-relaxation properties is another major strength of degradable hydrogels. By altering the molecular weight and density, hydrogel can mimic some of the dynamic mechanical properties of natural tissues under physiological conditions 15. For instance, hydrogels with adjustable stress-relaxation properties are developed by changing molecular weights to combine different calcium crosslinking densities, which crosslinks alginate ionically 27, or altering the molecular weight and density of polyethylene glycol (PEG) which is independent of the initial elastic modulus of the material 28. More novel preparation methods have been developed recently. In order to mimic the viscoelastic characteristics of bone ECM, Chen J et al. developed photocurable liquid crystal hydrogels based on chitin whiskers, and found negatively charged maleic anhydride chitin whiskers hydrogels were more conducive to the formation of bone than hydrogels based on positively charged chitin whiskers 16. Zhang J and his colleagues fabricated a kind of thermosensitive hydrogels, whose reversible mechanical deformation could be easily achieved through adjusting temperature from 25℃ to 37℃ 29. Upon sensing relaxation in the mechanical response, stem-cell spheroids promoted osteogenic differentiation by increasing the maturity of the FAs and the rate of F-actin polymerization 29. Moreover, the viscoelasticity of the hydrogel can also be dynamically changed by ionic cross-linking 30, improving hydrogen bond interactions 31 and hydrophobic interactions 32, 33.

A wide variety of other synthetic materials with excellent physicomechanical properties has also been explored. In contrast to the viscoelastic properties of hydrogels, studies on other synthetic matrix materials such as polymers mainly focus on the stiffness and surface nano-patterns design of materials, which are both valid parameters for regulating cell behavior 34.

Polydimethylsilane (PDMS) is a common polymer matrix material that can be easily fabricated into different stiffness 35, 36, which benefits the investigation on the specific mechanism of ECM stiffness to stem cell behavior. Changing the ratio of curing agent vs oligomeric base during substrate preparation is a common method to modify the stiffness of PDMS 35. Furthermore, it is also feasible to use temperature gradients to synthesize PDMS with stiffness gradients 36, or air plasma treatment to produce the desired wavy surface topology at different pressures and oxidation times 37.

PCL can also be synthesized with different stiffness 38. To explore osteogenic differentiation of MSCs on PCL scaffolds, multiwall carbon nanotubes (MNNTs) 34 and nano-HAp 39 are incorporated into PCL nanofibers to form a composite scaffold to enhance the material stiffness. The addition of functionalized MNNTs to PCL nanofibers independently changed the nanoroughness of PCL while adjusting its stiffness 34. In addition, the nacreous topology characteristic of the shell of invertebrates induced osseointegration and has been incorporated into the design of biomaterials 40, 41

Other methods of processing polymers to create micropatterns are usually based on reactive ion etching or multi beam laser interference on polyimide (PI) materials 9, and the microphase separation between poly(desaminotyrosyl-tyrosine carbonate) (PDTEC) and polystyrene (PS) 42. A poly(urea-urethane) nanohybrid scaffolds fabricated by 3D printing-guided thermally induced phase separation technique has the property of stiffness memory 43, and can be self-softening in a body temperature environment 44. In addition, superior physicomechanical properties have also been confirmed when natural biomaterials are combined with polymers, such as tissue engineering scaffolds with chitosan and gelatin combination 45 and silk fibroin combined with graphene oxide hydrogel matrix 46.

##### 2.1.1.2 Bioceramics

Bioceramic are widely used in BTE 47-49. Unlike hydrogels, bioceramic composites are poor in viscoelastic or stiffness tunable properties. Instead, they can mimic both physical architecture and chemical composition of nature bone 50, and be fabricated into different nanotopologies to regulate cell behavior.

As one of the most frequently used bioceramic materials in BTE 51, HAp can be processed in various forms and combined with a variety of other composite materials. The Ca(NO_3_)_2_·4H_2_O and (NH_4_)_2_HPO_4_ aqueous solutions can be treated by simple chemical precipitation to prepare nanosized HAp samples, and HAp nanorods of different shapes can be obtained by changing the reaction temperature and time, which show stronger osteo-inductive ability than traditional nano-HAp 52. Using this method, HAp nanorods which are similar to natural bone nanocrystals can be fabricated without organic solvents. The HAp micro-nanorod structure can also be loaded on the composite ceramic (β-TCP/CaSiO_3_) scaffold as a surface layer, and the process requires 3D printing technology 53. The strontium substituted HAp scaffold developed by Prabha RD et al. can be used to enhance alkaline phosphatase activity 54, an alternative processing modality for HAp. In recent years, researchers focus on the fabrication of surface topology. Ramaswamy Y et al. fabricated HAp surfaces with honeycomb, pillars and isolated islands topologies by microcasting with molds made of plant petals 55, which avoided the need for expensive micro-contact printing or lithographic devices and increases osteogenesis.

Recently, the composite scaffolds of bioceramics and other materials have also been extensively studied. Poly-l-lactic acid (PLLA)/HAp bone scaffold is prepared by enhancing the interfacial bonding between HAp and PLLA via nano-modifying HAp surface with a phosphonic acid coupling agent(2-Carboxyethylphosphonic acid) 50. In the latest study, PLLA coated with nanocomposite (NiFe_2_O_4_/ZnO) accelerated the osteogenic differentiation of MSCs 56. Composite scaffolds made of calcium-deficient HAp with fibrillated collagen and human umbilical cord serum (hUCS) have also been reported 57.

The surface nanotopology of other bioceramics such as silicon 58 and TiO_2_ 59 can be tuned to nanorod arrays. Moreover, BMP-2 coating can be added on TiO_2_ nanotubes 60. The behavior of the cells cultured on the surface of bioactive glass substrates nanorods was also similar to that of the cells on the hydrogel 61.

##### 2.1.1.3 Metal and alloys

Ti and Ti-based alloys have superior biocompatibility and osseointegration capability, playing an important role in the long-term survival of implants. Generally, bioactivity of the alloys is enhanced with the addition of bioactive elements, such as magnesium 62, cobalt-chrome-molybdenum 63, etc. Recently, surface modification has become a novel approach to accelerate the osteogenesis by improving the mechanical properties. The surface modification processes of Ti-based materials include sandblasting to change the roughness 64, 65, hydrofluoric acid etching to form micropitted topography 66 and hot solution of HCl/H_2_SO_4_ acid etching 67. In addition, the surface topology of pure Ti treated with hydrogen peroxide after acid etching was also shown to be favorable for bone integration 68.

Nanotopology are commonly fabricated in Ti alloy implants to drive osteogenesis. Ti-6Al-4V alloy with highly-ordered TiO_2_ nanotube structure stimulates the capacity of MSCs osteogenic differentiation. It is developed via electrochemical anodization, and the diameter of the nanotube can be adjusted by changing the voltage 69, 70,which is a processing method similar to that previously used for Ti 71. Pulsed laser remelting 72, femtosecond laser texturing 73, electron beam technique 74, and acid etching 75 have also been used to prepare Ti-6Al-4V alloy surface nanostructures. All of the above surface modification strategies enhanced the osteoinductive capability of alloy materials. In another study of other Ti-based alloys, the surface of Ti-25Nb-3Mo-2Sn-3Zr alloy treated by mechanical attrition treatment formed nanograined with osteogenic effect 76.

Tantalum (Ta) has unique advantages in promoting bone integration due to its good biocompatibility and mechanical properties 77. Chemical vapour deposition 78 combined with 3D-printing (selective laser melting) 79 can be used to process porous Ta, which has shown higher bone-induction ability than Ti-6Al-4V 79, 80. Ta alloys such as Ta-Ti gyroid scaffold 81 and Ti-Ta-Nb-Zr alloy 82 are also shown to upregulate the expression of osteogenic genes.

#### 2.1.2 Stiffness

Effects of substrate stiffness on regulating stem cell behavior has attracted significant attention in recent years 83** (Table 1)**. Engler AJ et al. demonstrated for the first time that matrix stiffness was a promising mechanical target to modify MSCs fate. In their study, MSCs were seeded onto collagen-coated PA substrates with three levels of stiffness. It was revealed that MSCs showed markers for neurogenic lineages on the softest gels (1 kPa), myogenic lineages at moderately stiff matrices (11 kPa) and osteogenic lineages at the stiffest matrices (34 kPa) 8. Interestingly, after several weeks of stiffness-directed differentiation, reprogramming of these lineages seemed to be impossible, even with addition of soluble induction factors. The stiffness-dependent differentiation has also been demonstrated in the study on human adipose-derived stem cells (hASCs). Hadden WJ et al. fabricated planar PA hydrogels with different stiffness gradients and analyzed stiffness-dependent hASC differentiation. Similarly, the expression of the adipogenic marker PPARγ peaked at low stiffnesses (E<3 kPa) after 6 days, MyoD, myogenic transcription factor, was highest around E∼12 kPa, and CBFA1, an osteogenic marker, was peak at E∼36 kPa 21.

Given that substrate stiffness exerts a significant influence on stem cell differentiation, researchers have started to perform a series of experiments to gain insight into its specific mechanism, and focus on exploring the optimal stiffness of biomaterials, to draw a feasible strategy for promoting osteogenic differentiation in BTE. Recently, numerous studies have confirmed that increased stiffness of biomaterials favorably drives stem cells into the osteogenic lineage 83-87. In the study of Liu Y et al., higher expression of differentiation markers in stiffer matrices demonstrated a more significant response of MSCs towards stiffer hydrogels. In contrast, the differentiation of MSCs in softer matrix appeared to be slower and more limited 86. This is because rigid substrates are more likely to induce F-actin polymerization and actomyosin cytoskeleton contraction, thus promoting nuclear translocation of YAP/TAZ and osteogenic differentiation of MSCs 88. Similarly, Zhang T et al. delivered straightforward evidence that rigid matrices allowed broader cell spreading, faster cell growth and stronger expression of vinculin in ADSCs 85. This might because viscoelastic behavior presented by low stiffness influences cell spreading and stromal cells fate 85.

It has previously been shown that osteogenic differentiation of MSCs mainly occurs at 25-40 kPa 8. Interestingly, MSCs can also respond to stiffness beyond this range. Yang Y et al. manufactured polyethylene glycol/silk fibroin/hydroxyapatite (PEG/SF/HAp) scaffolds with different proportions of HAp (25, 50, 75, and 100 mg), and the stiffness ranged from 80.98 to 190.51 kPa. The results showed that scaffolds with 50mg HAp (nearly 130 kPa) significantly enhanced the effect of osteogenesis, compared with the stiffer or the softer ones 89. However, when the stiffness reaches 600-700 kPa, cellular growth and osteogenic differentiation was more obvious 90. And lower stiffness presents better osteogenesis when stiffness lies outside of this optimal range 26. Hu Q et al. manufactured demineralized bone matrix (DBM) scaffolds with various compressive modulus (66.06 ± 27.83 MPa, 26.90 ± 13.16 MPa and 0.67 ± 0.14 MPa). In contrast to the two former ones, DBM scaffolds with a stiffness of 0.67 ± 0.14 MPa promoted osteogenesis, and significantly enhanced bone integration 91. Similarly, Maggi et al. constructed 3D nano-structured scaffolds with stiffness ranging from 0.69 ± 0.2 MPa to 60.2 ± 7.4 MPa. They found that the nanolattice with lowest stiffness (0.7 MPa) exhibited 20% more F-actin than others 92.

There are several potential mechanisms that may explain why there are some biomaterials with less stiffness perform better in supporting cell proliferation and enhancing osteoblastic differentiation. On one hand, integrins bond formation between MSCs and soft matrix is higher than in the stiff one, which in return promote MSCs osteogenic differentiation 26. On the other hand, degradation-mediated cellular traction is another essential element to regulate the differentiation of MSCs 26. In stiff scaffolds with high alginate concentrations, cell-mediated degradation may be slow, resulting in low traction between the cell and substrate, thereby inhibiting osteogenesis. Conversely, the cell-mediated degradation in soft substates exhibits a high degree of cell diffusion and high traction, which favors osteogenesis 26. Additionally, for high substrate stiffness, the cell's ability to sense biophysical cues in the microenvironment is reduced, preventing excessive mechanical signals from being transmitted to related proteins on the cell membrane, resulting in reduced osteogenic differentiation ability 89.

#### 2.1.3 Viscoelasticity

How does matrix elastic modulus/stiffness affect cell-matrix mechanical interactions and MSCs differentiation has been extensively studied through researches on elastic biomaterials 83, 93. It should be noted, however, that the ECM of bone tissue is not purely elastic, but viscoelastic 16. The resident cells sense and respond to the mechanical deformation caused by viscoelasticity in a time-dependent manner 96. Recently, it has been demonstrated that the viscoelasticity of bone ECM plays an essential role in regulating cell behaviors and osteogenic differentiation 16. Therefore, how to better simulate the viscoelasticity of bone ECM is crucial for the design of scaffolds in BTE 16.

Beyond the characteristics of elastic solids, more importantly, biomaterials with viscoelastic properties need to contain the characteristics of viscous fluids 7, 97. The elastic properties determine its elasticity as well as the initial resistance to applied forces 5. Nonetheless, biomaterials with elasticity solely restrict cell adhesion, proliferation, diffusion and differentiation to a large extent because of their inability to relax forces effectively 98, 99. In contrast, the viscous properties dissipate the applied load and lead to extinction of drag force as well as permanent deformation over time.

During this process, the stored energy is fully released through stress relaxation. This stress release not only guides cells to reshape the matrix, but also transforms a dynamic signaling within stem cells that regulates its spreading, polarization and differentiation in turn 5, 100, 101.

In recent years, a growing effort has been devoted to developing viscoelastic substrates with stress relaxation to regulate osteogenic differentiation by simulating the mechanical microenvironment of bone tissue 7, 99, 102** (Table 2)**. Hydrogels are considered to be the promising candidates for simulating bone ECM due to their highly adjustable biophysical properties 103. Chaudhuri O et al. developed a synthetic hydrogel system for the first time to simulate the stress relaxation behavior of viscoelastic tissues. It was demonstrated that osteogenic differentiation of MSCs changed with alterations to the matrix viscoelasticity, and significantly increased when cultured in a substrate with faster relaxation kinetics, compared with a static substrate 27. It is possibly due to rapid stress relaxation regulates intracellular integrin adhesion and actomyosin contraction, as well as nuclear localization of mechanosensitive transcriptional regulator YAP, thereby promoting osteogenic differentiation of MSCs 27. It has been further demonstrated in follow-up studies that, except for the direct osteogenic action on stem cells, fast relaxing matrices facilitates bone matrix formation by stimulating cell volume expansion, adhesion, spreading and proliferation as well 28, 97, 102, 104, 105. These results suggest that bone formation capacity of biomaterials can be optimized by adjusting the stress relaxation timescale and thereby changing the viscoelasticity of the matrix 7, 104.

Although several researches on regulating matrix viscoelasticity have been reported, these methods usually require complex physical crosslinking methods and chemical treatments 99, 106-108, and they merely focused on improving the viscoelastic properties of the material itself to achieve a high level of bone regeneration. Future directions need to focus on the new possibilities of combining with strategies that facilitate bone regeneration, such as stem-cell spheroids 29, 30, the addition of natural ECM 109, etc. Moreover, additional in vivo analytical models are required to investigate changes in viscoelastic properties at the bone-implant interface, in order to accurately predict the degree of bone integration 110. Only in this way can appropriate biomaterial systems be constructed to better simulate the viscoelasticity of bone tissue ECM as well as guide the function and fate of stem cells.

#### 2.1.4 Topography

As is well-known, superior mechanical properties of biomaterials is regarded as one of the evaluation criteria of medical implants 55. Different from the modulation of stiffness and viscoelasticity, the topological structure printed on the substrate surface has greater clinical translational value due to its negligible effect on the overall mechanical properties of the material 55. Moreover, altering surface topography gains popularity for offering not only the advantage of long-term stability, but also cost-effective fabrication methods 111. Ever since Harrison RG et al. first confirmed in 1911 that stem cell differentiation could be modulated by topographic cues from underlying substrates 112, considerable effort has been devoted to guiding the MSC lineage determination by adjusting the surface topology of materials **(Table 3)**.

As an important feature of surface topography, the roughness of biomaterials can directly regulate the migration and proliferation of cells on the surface 113. More importantly, compared with smooth surface, rough surface topology enables stem cells with better osteogenic capability 114. For instance, Yang W and his colleagues fabricated HAp-based scaffolds with different surface roughness. It was found that scaffolds with average roughness (Ra) (0.77 -1.09 μm) and mean distance between peaks (RSm) (53.9 - 39.3 μm) achieved optimal osteogenic differentiation by influencing cell attachment and cytoskeletal tension 115. However, it should be noted that, surface topologies with different roughness can also induce the adipogenic differentiation of stem cells. Abagnale G et al. discovered that 2μm ridge enhanced osteogenic differentiation, while 15μm ridge supported adipose differentiation. This may be attributed to the direct effect of their physical size on cell morphology, with elongated morphology promoting cell progression toward osteoblastic lineages and rounded morphology promoting lipogenesis 9. Recently, various rough topographies (such as ribbon structures 42, wavelike structures 37, 116, groove or ridge structures 9, microchannels 117, isolated islands 55, etc) have been successfully fabricated to promote osteogenic differentiation, among which the ribbon structure is the most widely used 6. Vega SL et al. fabricated substrates with co-continuous (ribbons) or discontinuous (islands and pits) regions. The findings show that ribbon topographies (spacing: 48±5μm) favor cytoskeletal anisotropy and FA maturation, which promoted long-term expression of osteogenic differentiation markers 42.

Aside from the micron-structured biomaterials mentioned above, the interaction between cells and nano-morphology is also considered to be an effective approach to control stem cell differentiation in BTE 59. This is because bone itself has the unique hierarchical nanostructure structure 118. TiO_2_ nanotube arrays, manufactured by anodizing on a Ti substrate, are most commonly used to investigate the effects of nanoscale geometry on stem cell behavior 118, 119.

The early experiments proposed that the difference of diameter gave rise to different mechanisms responsible for osteogenic differentiation of stem cells 120. Small diameter (approximately 30 nm) nanotubes promoting cell adhesion, conversely, larger diameter (70-100 nm) ones benefited cell elongation, which might lead to a change in cytoskeletal stress 120. Recently, Lv L et al. found that TiO_2_ nanotubes, whose diameter were 70nm, were the optimal size for osteogenic differentiation of hASCs, compared with that of 50nm and 100nm 118. Similarly, the optimal osteogenic diameter of nanorods was also confirmed to be 70nm 121. In addition to the diameter, the distance between nanotubes also has implications on cell differentiation. Smaller pitch promoted MSCs differentiation from a young donor, while a larger pitch promoted that from an old one. This suggests that the nanotube spacing can be adjusted according to the age of the patient to prepare novel implants with the best osteogenic effect 58. Nanogrooves and nanofibers are also proved to be powerful for material-driven osteogenesis. Yang L et al. combined substrates with nanogrooves and cell-derived matrices (CDM), which dramatically enhanced osteogenesis. However, CDM itself displayed only a minor contribution without nanogrooves. This suggests the strong synergistic effect on MSC osteogenesis 116. Another combinatorial scaffold system was established by utilizing nanofiber scaffolds and polymeric microspheres. The nanoscale fibers not only mimic natural ECM, but also evoke directed response, especially osteogenesis 122.

Nevertheless, Li X et al. found that nanostructures alone might not be the optimal structure for osseointegration. Compared with flat quartz, nano-morphology significantly abated the osteogenic capacity 61. Furthermore, the structural size of stem cells and natural ECMs is usually at the microscopic level. Stem cells may fail to stimulate osteogenic developmental signaling pathways due to their inability to perceive nanotopology 6. Therefore, increasing researchers have recently devoted themselves to developing biomaterials with micro/nano-scale hybrid topologies, which show excellent osteogenic effects 123-125. In fact, the pro-osteogenic mechanism of micron and nano-structure is different 126, 127. Micro-groove promotes stem cell differentiation by activating the formation of pseudopodia. In contrast, nano-groove stimulate the formation of FAs and activates the RhoA/ROCK pathway, which shows stronger effects on osteogenesis 126. It was further confirmed that the two structures have different activation mechanisms for integrins 127. Therefore, the combination of microstructures and nanostructures has a synergistic activation effect 127.

To faithfully represent the in vivo-like microenvironment with complex topological structure, nacre topography with better osteoinductivity was fabricated via biomimetic approaches 128, 129. It was shown that bone tissue that formed in response to nacre topography exhibited a higher crystallinity than those to chemical cues 128. Furthermore, other biomaterials with novel topologies have been shown to be osteoinductive, such as randomly oriented fiber scaffolds 130, quartz with chiral geometry 131, multiscale hierarchical topography 132, etc. Nonetheless, the most current surface morphologies are designed on plane models. The construction of biomaterials with 3D topological structures is an urgent issue in the process of clinical transformation 59.

#### 2.1.5 Dimensionalities of internal mechanical stimulation

Recently, dimensionality has been demonstrated to be a major contributor to affect cellular responses to mechanical stimulation. Vastly different outcomes have been shown when cells are cultured in 2D versus 3D microenvironment 133. Generally, dimensionalities alter cellular shapes, thus affecting cell proliferation, migration and differentiation. It was shown that cell shape was flatter in 2D than in 3D, which might be related to whether integrin-mediated cell adhesion occurs on one side or around the cell, thereby influencing F-actin arrangement and expression 134.

Although researches on 2D culture are well established, 3D cell culture platforms have attracted attention recently, for mimicking more closely the geometrically complicated environment in vivo. Since the cells in 3D microenvrionnment may be affected by material stiffness, topography, permeability, oxygen, and other factors, a separate study on dimensionality appears to be unrealistic. Hsieh W-T et al. investigated the influence of dimensionality and stiffness on osteogenic differentiation of MSCs 135. The results showed that the cell differentiation capability of 3D scaffolds was significantly enhanced with the increase of stiffness, compared with 2D substrates. This is due to the increased abundance and good alignment of actin stress fibers in a 3D environment with high stiffness. However, Major L G et al. held the opposite viewpoints 133. They maintained that the cells responded to stiffness in a totally different way in 2D and 3D environments. With the increase of stiffness, cell volume increased in 2D environment, while, an opposite trend was observed in the 3D environment, along with decreased expression of the osteogenic gene RUNX2. This might due to the physically restriction to cell volume in 3D microenvironment 133. To further explore the effect of cell volume on osteogenic differentiation, Bao M et al. developed a way to change cell volume alone, instead of depending on stiffness in a 3D microniche. It was shown that in small cells, stress fiber formation and YAP/TAZ localization could be observed on both soft and stiff matrix, showing that the osteogenic differentiation of cells was not affected by stiffness in cells with small volume. Conversely, stiffness was the major determinant for stress fiber formation in the largest cells 22. This finding suggests that the difference brought by dimension (to be more specifically, physically restriction to cells) should be taken into account when designing biomaterials with various stiffness in the future.

In addition, dimensionality can also affect the optimal oxygen content of MSCs in scaffolds, thus affecting their osteogenic differentiation. It was shown that the expression of RUNX2 and VEGFA reached the highest when O_2_ concentration was 5% in 2D environment, while in 3D environment, O_2_ concentration needed to achieve up to 21% 136. However, the reasons for this difference remain to be studied. In conclusion, dimensionality alters cellular response to biomaterial properties to some extent, thus affecting osteogenic differentiation, but the underlying mechanism by which mechanical stimulation regulates cell fate in different dimensionalities requires further exploration.

### 2.2 External mechanical stimulation on MSCs-laden biomaterials

Numerous studies have demonstrated that mechanical properties of biomaterials promote the osteogenic differentiation of MSCs. However, static culture may lead to insufficient supply of nutrients and oxygen 2. Conversely, dynamic culture allows for more adequate nutrition and is closer to physiological systems in vivo, thus showing better osteogenesis 2. Therefore, the application of various mechanical stimuli by bioreactors, such as shear stress 137, and micromechanical strain induced by compression, tension and vibration 138-140, becomes a promising approach to induce osteogenic differentiation of MSCs in vitro 141 **(Table 4)**.

#### 2.2.1 Compressive stress

Physiologically, the bone matrix is subjected to compressive or tensile loading due to gravity and muscle contraction 147. This mechanical stimulation acts on the cells in the bone tissue and plays an important role in bone remodeling, such as early bone healing when fractures or bone defects occur 2. Therefore, in order to investigate the optimal compressive stress on osteogenic differentiation of MSCs in vitro, large numbers of studies have been conducted by using compression bioreactors 158, 159.

Compared with 2D environment, MSCs loaded on 3D scaffolds are studied more extensively in recent years, which is more closely to the physiological conditions in vivo 2. It was found that compressive stress could promote osteogenic differentiation of MSCs in octacalcium phosphate-gelatin scaffold under a certain stress amplitude (20%) 143. However, excessive stress amplitude (40%, 60%) inhibited the differentiation of MSCs. These results indicated that stress amplitude had significant effect on MSCs differentiation. In addition, compressive stress can also promote the differentiation of MSCs indirectly by altering the stiffness of scaffolds. In the study of Baumgartner W et al., it was found that under the condition of 5% cyclic compression, the stiffness of PLGA/aCaP scaffolds increased by about 2 times, and osteogenic markers RUNX2 and type I collagen were significantly up-regulated 160.

Compressive stress acts on the scaffold material and is then delivered to the cell 161. Therefore, the mechanical stimulation of compression sensed by cells is related to the scaffold material. Hydrogels are often used as cell-loaded scaffolds in compression bioreactors because of their low elastic modulus and no noticeable deformation even in the setting of repeat compression forces 159. The effects of different concentrations of GelMA hydrogel (5%, 7.5%, 10%) and dynamic compression (0, 10, 27 and 42%) on cell differentiation were studied by Seo J et al 140. The results showed that 5% GelMA hydrogel and 42% dynamic compression had the best effect on cell diffusion and osteogenic differentiation, with the overexpression of ALP, OCN, OPN and mineral deposition. The reason may be that the degree of crosslinking of hydrogels affects the size of pores in the polymer network. The higher the degree of crosslinking, the smaller the pores, resulting in reduced cell diffusion and growth, which affects the transmission of compressive force 140. Therefore, hydrogels with lower degree of crosslinking provide larger pores and promote cell migration, which is more recommended.

However, counter to the view as mentioned above, some experts hold that dynamic compression stimulation could not significantly promote the osteogenic differentiation of MSCs. It was found that cyclic compression stimulation (5%, 10%) reduced MSCs migration, but did not stimulate osteogenic differentiation. Meanwhile, the up-regulation of transcription factor RUNX2 should be followed by the up-regulation of BMP-2 142. It was also found that dynamic compression (15%) was more conducive to chondrogenic differentiation of MSCs 139. Therefore, the magnitude and mechanism of appropriate compressive stress promoting osteogenic differentiation of MSC need further investigation.

#### 2.2.2 Tensile stress

Distraction osteogenesis is a treatment modality applied to the healing of bone defects 162. It stimulates new bone production by stretching the fractured end toward the other end. Therefore, it is suggested that loading cells with tensile stress using a tensile strain bioreactor promotes osteogenic differentiation of MSCs. Qi et al. investigated the effect of short-term tensile stress (0.5 Hz, 2,000 με) on the proliferation and osteogenic differentiation of MSCs 163. The expression of growth factors TGF-β, bFGF and IGF-II and transcription factors RUNX2 and Ets-1 were upregulated under the stress. Wu et al. also found that short-term tensile stress (10%, 0.5Hz,6h/day) promote the expression of OPN, RUNX2, and OCN 164. And this study further found that long non-coding RNA H19 was a positive regulator in osteogenesis of MSCs. It indicates that tensile stress has a critical role in promoting osteogenic differentiation in MSCs.

In order to fully simulate and investigate the effect of tensile stress on MSCs in vivo, cell-laden 3D biological scaffolds are fabricated recently. The hydrogel-coated MSCs, stimulated by uniaxial cyclic stretching, was found to promote osteogenesis and the expression of TNC markers 144. Meanwhile, MSCs differentiation is dependent on the frequency and amplitude of strain in the endochondral osteogenic pathway. The expressions of osteogenic markers BMP-2, RUNX2, OPN and COL3A1, and chondrogenic genes ACAN and SOX9 were more strongly expressed at high amplitude and frequency (10%, 1 Hz) than at low amplitude (5%) 144. It shows that the osteogenic pathway can be activated by adjusting the frequency and amplitude of tensile stress. However, studies on the effect of tensile stress on osteogenic differentiation of MSCs in 3D scaffolds are still relatively few, and more studies are needed in the future to explore the optimal tensile stress and the involved mechanic pathways.

#### 2.2.3 Fluid shear stress

Many studies have shown that pretreatment of MSCs in a bioreactor promotes new bone formation in vivo 154. Fluid shear stress in vivo can be simulated through a perfusion bioreactor, which facilitates osteogenic differentiation. Fluid shear stress enables cells seeding in a dynamic fashion, allowing them to be more uniformly distributed inside the scaffold, rather than just being located on the surface of the scaffold 141. Cells located on the surface of the scaffold are easily washed away under high shear stress, which greatly reduce cell viability. At the same time, compared to static cell culture, the perfusion bioreactor drives the flow of medium at a certain rate, which facilitates the provision of more adequate nutrients and oxygen to the cells inside the scaffold, transports metabolic wastes, and maintains cell viability 150.

Based on the above-mentioned advantages, fluid shear stress has been extensively studied in terms of promoting cellular osteogenic differentiation 146. Since the fluid shear stress to which the cells are subjected is generated by the perfusion device and transmitted through the scaffold, the effect of MSCs osteogenic differentiation is closely related to the appropriate perfusion conditions and culture medium, as well as the physicomechanical properties of scaffolds 165. In recent years, an extensive investigation has been conducted into the effects of flow rate and incubation time on MSCs osteogenic differentiation under laminar, radial, and oscillatory fluid flow (OFF) through various perfusion bioreactors 154. Laminar flow, which is unidirectional perfusion, provides a mild culture environment for cells and has been shown to promote MSCs osteogenic differentiation when cultured dynamically at low flow rates in normal medium 148. In the study of Yamada S et al., it was confirmed that even in the absence of chemical stimulation, fluid stimuli in appropriate level significantly promoted osteoblastic differentiation of MSCs on 3D scaffolds 148. Oscillatory perfusion helps to distribute the cells more uniformly within the scaffold 147. According to recent studies, MSCs were cultured in osteogenic induction medium supplemented with dexamethasone, β-glycerophosphate and ascorbic acid 2-phosphate to further enhance osteogenic differentiation 149. However, there are certain differences in the appropriate perfusion velocity and culture time required by different devices and scaffolds. Generally, the fluid shear stress that between 0.1 and 10 MPa is considered to promote bone tissue regeneration 51. Excessive shear stress has a damaging effect on cells 148. However, in the study of Mainardi VL et al. 146, the fluid shear stress provided by the high perfusion flow rate of 7ml/min (56.09 MPa) was slightly higher than this range, but compared with the low perfusion flow rate of 0.7ml/min (5.59MPa), the effect of alginate-gelatin hydrogel scaffold on promoting the deposition of mineralized matrix was more obvious. The reason might be that cells were embedded inside the scaffold by 3D bioprinting technology, and the fluid shear stresses that received were converted more into compressive and tensile mechanical stimuli, thus promoting the deposition of mineralized matrix. Similarly, in the study by Yaghoobi M et al. 154, high flow rate (4.5 ml/min), compared to low flow rate (2 ml/min), promoted the upregulation of RUNX2 expression in MSCs in nHA-PCL multilayer electrospun silk scaffolds under the conditions of combined mechanical pressure. Thus, higher perfusion flow rate shows better osteogenic potential.

In terms of culture duration, continuous perfusion culture tends to reduce the cellular response to stress stimuli and make cells adaptive 166. Therefore, intermittent perfusion culture is more favorable. A culture time from 5 days to 5 weeks is the most widely-applicable 167. However, some studies have shown that longer culture time does not necessarily imply better osteogenesis. MSCs produced the highest angiogenic markers at 7 days under perfusion culture at a flow rate of 3 mL/min, but the DNA content and osteogenic differentiation markers stabilized at 14 days 168. Suzuki K et al. used a different culture protocol, using standard medium for one week and then osteogenic differentiation medium for one week 149. The highest ALP activity was observed at this time point, however, the activity of ALP decreased at 2 weeks of osteogenic differentiation medium culture. It indicates that MSCs may promote early bone differentiation. Therefore, it is important to find the appropriate culture time for different materials.

In addition, the fluid shear stress is closely related to the pore size of the scaffold 51. It is generally believed that a pore size of over 300 μm is favorable for cell migration, proliferation, and the growth of blood vessels and bone tissue into the scaffold 169. It was further demonstrated that the shear stress provided by the medium pore size (750-900 μm) of the HAp scaffold (2.65 MPa) was more suitable for osteogenic differentiation compared to the large pore size (1.55 MPa), and the small one (5.78 MPa) 51. This suggests that when exploring the optimal scaffold pore size, the effect of shear stress also need to be considered. Moreover, in the study by Rogina A et al., chitosan scaffolds containing 30% HA showed significantly higher deposition of collagen and calcium after 21 days of perfusion culture compared to 50% HA versus chitosan scaffolds alone. This indicates that besides pore size of scaffolds, the chemical composition also influences cell adhesion, growth, proliferation and differentiation 153.

#### 2.2.4 Vibration

Low-magnitude, high-frequency vibration plays a critical role in maintaining bone homeostasis and promoting bone metabolism, and was recently introduced to induce MSCs osteogenesis 170, 171. Prè D et al. studied the effects of high frequency vibration (30 Hz, 0.59 × g, 45 min/day) on the proliferation and osteogenic differentiation of MSCs and found that calcium deposition, type I collagen deposition, and RUNX2 expression were significantly increased after 21 days of culture 170. Another study showed that vibratory stimulation (50 Hz, 0.05-0.9 × g, 30 min/day) promoted osteogenic differentiation of periodontal stem cells 171. These results suggest that MSCs respond to the mechanical effects of high-frequency vibration and can be induced to osteogenic differentiation by loading cells with high-frequency vibration.

Vibrational bioreactor, an in vitro device that generates high frequency vibrations, is extensively studied in recent years, especially nanovibrational bioreactor. The nanovibrational bioreactor allows the culture to produce nanoscale displacement at a certain frequency to induce osteogenic differentiation of MSCs 138. However, in 3D scaffolds, nanoscale vibrations need to be transmitted through the scaffold to the cells, thereby making stable transmission of vibrations more difficult. Because of the good viscoelastic characteristics, type I collagen gel can adhere to the sidewalls and bottom of the culture dish, forming a monolith with the culture vessel and good delivery of vibrational stimuli 156, which makes it more commonly used in the study of nano-vibrational bioreactors 172. It was found that the expression of osteogenesis-related genes such as ALP, OCN and OPN were significantly increased in collagen gels under nanoscale vibration (30 nm, 1 kHz) using the principle of reverse piezoelectricity 156. It indicates that nano-vibration stimulation has a positive effect on MSCs osteogenic differentiation. Meanwhile, it was further revealed that nano-vibration stimulation could be delivered to cells via mechanoreceptors such as Piezo, TRP and potassium channel subfamily K member (KCNK), affecting cytoskeletal tension and adhesion. However, the mechanism of nano-vibration stimulation on cells in 3D scaffolds is still unclear. Therefore, the osteogenic differentiation of MSC under 90 nm amplitude conditions was further investigated 157. It was found that the expression of mechanoreceptors TRPA1, Piezo1/2 and KCNK2 were upregulated in cells at 90 nm amplitude compared to 30 nm amplitude. Thus, higher nano-amplitude showed a greater advantage in 3D scaffolds. However, higher amplitudes are associated with higher levels of reactive oxygen species and inflammation, which inhibit osteogenic differentiation of cells 173. Therefore, it is important to balance both osteogenic differentiation and inflammation levels when designing the amplitude.

### 2.3 Combined effects on MSCs-laden biomaterials

It is well known that cell behaviors are profoundly affected by the variable 3D surrounding microenvironment. Hydrostatic pressure (HP), fluid shear stress (FSS), compression, and stretching mechanical stimulation work together with the complex structure of ECM to affect cell fate 174. Therefore, it is believed that researches on the combined effects of multiple physical cues can better simulate the microenvironment in vivo, thus promoting the osteogenic differentiation of cells 175. Reinwald Y et al. confirmed that intermittent hydrostatic pressure (IHP) (270 kPa) in combination with topographical cues (fiber alignment) could direct the fate of MSCs, and enhanced the effect of osteogenesis 174. Moreover, cells on random fiber substrates were more responsive to the IHP, compared with those on aligned substrates 174. Two types of FSS, uniaxial rotation and perfusion were combined to investigate the effect of MSCs osteogenic differentiation in 3D β-TCP scaffolds 176. The results showed that the rotated and perfused group significantly up-regulated ALP activity and the expression of OCN, RUNX2, and COLI, compared to perfusion alone. The combination of FSS and compression was also explored. Ravichandran A et al. invented a biaxial rotation bioreactor that rotated along the X and Z axes, similar to the gyroscopic motion of a fetus in utero, and is simultaneously loaded with cyclic compression stimuli to mimic the biomechanical stimulation to which bone is physiologically subjected 155. It was shown that the biaxial rotation approach increased the rotational velocity component compared to the uniaxial rotation, thereby improving fluid transport within the scaffold. The expression of RUNX2 and COL1A1, as well as mineral matrix deposition, were significantly elevated after simultaneous loading of cyclic compression stimuli.

As additional insights into the interaction between cells and ECM in recent years, it was found that the combination of matrix stiffness and nano- topography significantly affected the fate of cells as well 175. After attaching to the random nanofibers, cells presented apparent stretching morphology and transformed mechanical signals into intracellular signals through cytoskeletal rearrangement, thus promoting osteogenic differentiation 174. And this effect could be amplified by the combination with matrix stiffness 175. Nevertheless, it is difficult to obtain the optimal stiffness and topography for osteogenesis simultaneously. Jahanmard F et al. attempted to investigate the balance between stiffness and topography by adding carbon nanotubes to the substrate 34. It was found that low concentration of carbon nanotubes (0.5wt %, 1wt %) significantly improved the stiffness of electrospun nanofibers, while relatively high concentration (2wt %, 3wt %) showed obvious nano roughness. Moreover, the two above-mentioned mechanical forces show distinct effects on MSCs. High stiffness promotes cell proliferation and osteogenic differentiation, while roughness affects cell morphology and cell adhesion 19. Seo J et al. further demonstrated that stiffness increased the number of adhesion sites on MSCs, but for mature adhesion sites, it was only determined by the roughness of surface topography 140. At present, although the influence of matrix stiffness and surface topography on osteogenic differentiation of MSCs has been understood to a certain extent, how to balance the relationship of two for enhancing the synergistic effect and better promoting osteogenic differentiation remains to be further studied.

## 3. Mechanisms involved in biomaterials-induced osteogenic differentiation of MSCs

### 3.1 Mechanosensors

**Figure 2:**
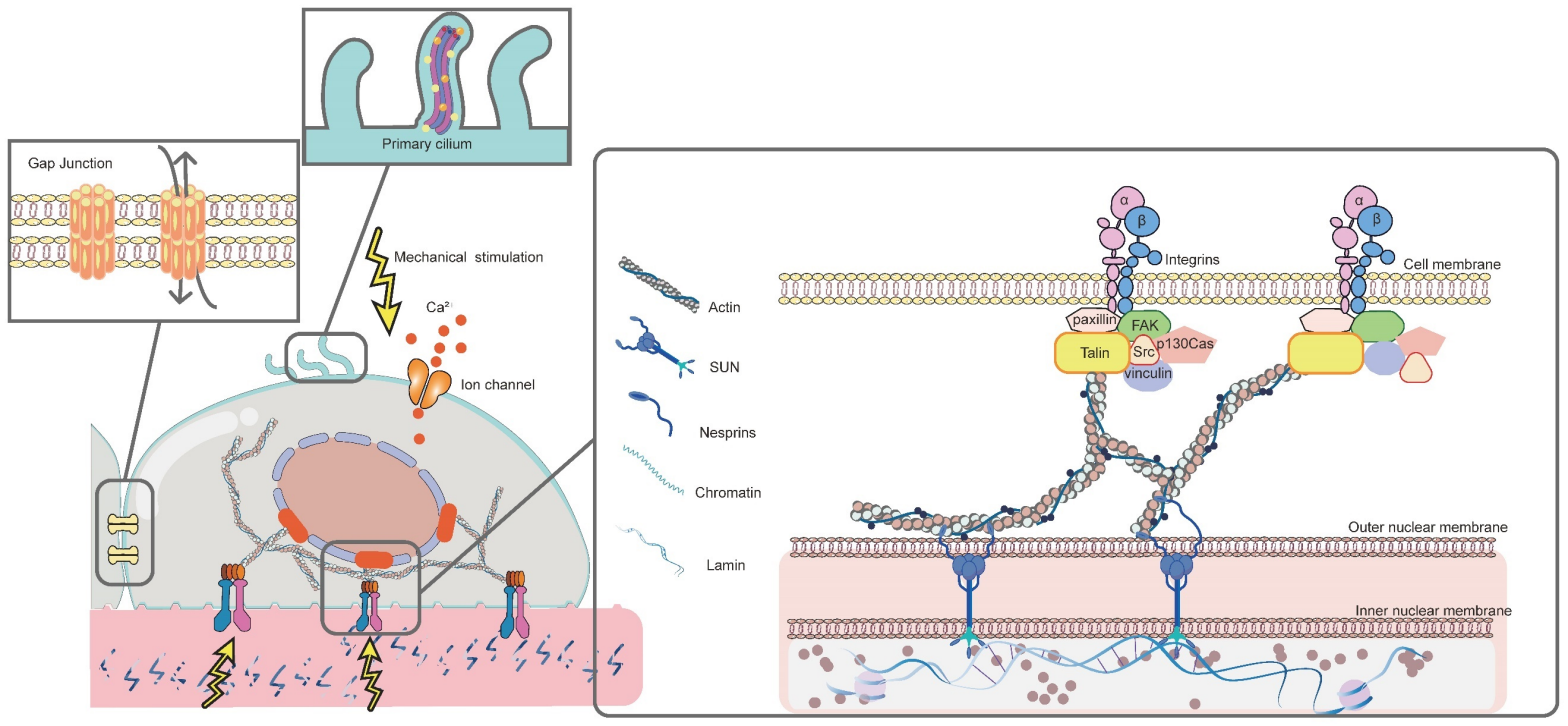
Mechanosensors involved in biomaterials-induced osteogenic differentiation of MSCs. Mechanical stimulation is sensed by different mechanosensors on MSCs, including integrin and FAs, cytoskeleton, primary cilium, ion channels and gap junction. FAs function by transmitting mechanical signals to the cytoskeleton, thereby affecting cytoskeletal arrangement. Primary cilium alters the length in response to mechanical signals. Ion channels permit Ca^2+^ influx to modulate downstream pathways. Gap junction mediates cell-cell interactions by upregulating the expression of osteogenic-related genes.

The response of MSCs to mechanical stimulation comprises two major phases: mechanoreception and mechanotransduction 3. Mechanoreception is the process that cells sense physicomechanical signals from the ECM through mechanoreceptors. This further leads to cell differentiates into specific lineage through signaling pathways, which is known as mechanotransduction 3. Mechanoreception is crucial for transforming physical signals into biochemical ones by adjusting cytoskeletal arrangement, cell and nucleus morphology 177. Herein, the major mechanosensors, including integrin and FAs, cytoskeleton, primary cilium, ion channels, and gap junction, will be discussed in detail **(Figure 2)**.

#### 3.1.1 Integrin and focal adhesions (FAs)

Integrins, widely known as mechanical sensors, are ubiquitous in thin cell membrane projections and filopodia, mediating adhesion between cells and ECM and transmitting mechanical signals 55, 178. As a transmembrane protein, one end of the integrin is attached to a protein ligand in ECM, the other connects to the intracellular actin fibers via an adaptor protein 3. This establishes an integrin-dependent bidirectional signaling, that is, not only transmiting cellular signals to the ECM, but also conveying signals from the ECM intracellularly can be achieved 179, which triggers intracellular signaling pathways that lead to cell migration, proliferation, and differentiation 127.

Integrins are heterodimers, consisting of non-covalent binding of α and β subunits. Compared to β-subunit integrins promoting intracellular signaling and cytoskeletal linkage, the α-subunits induce ECM ligand specificity 13. In vertebrates, the 18 α and 8 β subunits assemble 24 complexes with diverse functions 180. Different subunits play distinct roles in regulating stem cell responses to the physicomechanical properties of the microenvironment. During the osteogenic differentiation of MSCs, the expression of integrins α1, α2, α5, αv, and β1 are upregulated 125, 181-184, and α3, α4, α6, β2, β3, β4 are downregulated 185, 186. Interestingly, the expression of integrin on cell surface fluctuates with different types of mechanical stimulation. For example, more α5β2 is expressed on microstructures than on nanostructured materials, while there is no significant difference in the expression of αvβ2 on microstructures and nanostructures 127. High matrix stiffness promotes α2 integrin expression 183. Furthermore, when binding to various ECM proteins, subunits show different affinity and specificity. For instance, mediated mechanical transduction mediated by fibronectin requires the synergistic action of multiple integrin subunits such as αvβ3, α2β1 and α5β1, whereas type I and type IV collagen require only α5 integrins 187. This may be due to the fact that the arginine-glycine-aspartic acid (RGD) motif of fibronectin needs to recognize the epitopes of two subunits, whereas the binding site of collagen only needs to bind to the specific domain of the α subunit 177.

In response to mechanical stimulation, integrins with higher affinity are activated, enabling the recruitment of a wide range of intracellular proteins, which are termed as integrin adhesion complexes (IACs). They mediate mechanical signals between the integrin and actin cytoskeleton, which further directs stem cell differentiation 188. Three protein layers assemble the IACs. The outermost signaling layer contains highly phosphorylated proteins FAK and paxillin. The intermediate force transduction layer consists of two adaptor proteins, talin and vinculin. And the innermost actin regulatory layer is dominated by α-actinin 189, 190. It is worth noting that talin plays a major role in IACs. This encoded protein forms the integrin-protein-actin axis complex by coupling integrin to F-actin 177. Following the transfer of mechanical forces from the integrin, talin transitions into an unfolded conformation and exposes its binding site 190, allowing IACs to rapidly aggregate into focal complexes and further mature into supramolecular complexes known as FAs in a brief period of time 177.

The physicomechanical properties of different biomaterials directly influence the number and size of FAs, which significantly affect the osteogenic differentiation of MSCs 13. For example, enhanced matrix stiffness increases the expression of specific integrins (αv, α5, and β1), inducing the formation of FAs and the further activation of downstream osteogenic signaling pathways 184. Additionally, the area of FAs increases with the roughening of material surface 14. Compared with a flat substrate, the 100μm groove/ridge promotes mature FAs formation, leading to osteogenic differentiation of MSCs. Conversely, the 10μm groove/ridge array formed fewer FAs and promoted adipogenic differentiation 191. Two potential reasons can explain why mature FAs drive osteogenesis. On one hand, they can directly transmit mechanical signals to sensors for nuclear mechanics (lamin A/C) through actin stress fibers 192. Specifically, FAs modulate the spatial organization of radial and transverse fibers in actin cytoskeletons. In this way, the FA-nuclear mechanical coupling is established, and physical signals are translated into biological activities controlling MSC fate commitment 193. On the other hand, FAs induce downstream cell responses through chemical signals, involving the recruitment and activation of signal proteins, dominated by FAK 14. Under mechanical stimulation, the conformation of FAK changes, exposing phosphorylation sites and activating intracellular osteogenic-related pathways 14. Moreover, other key signals related to osteogenic differentiation are also activated, such as BMP 127, RhoA 55, extracellular-signal-regulated kinase (ERK) 181, etc. It is thus clear that abundant and tightly packed FAs are essential for subsequent cytoskeletal changes and the triggering of intracellular signaling pathways during physicomechanical stimulation-induced osteogenesis.

#### 3.1.2 Cytoskeleton

The cytoskeleton, including the cytoplasmic and nuclear skeleton, is responsible for maintaining cell shape, motility, contractility, etc. More importantly, they also act as mechanosensors of ECM 13, 194. The cytoplasmic skeleton senses mechanical stimulation in ECM and then transmits signals to the nucleus, ultimately alterating the gene expression 10. The structural elements of the cytoplasmic skeleton are composed of microfilaments, microtubules, and intermediate filaments (IFs) 195. Among them, the actin microfilaments play a critical role in transmission of mechanical signaliing, which are tightly connected with FAs and nucleus 195.

Acting as a highly dynamic network, actin cytoskeleton realizes the transmission of mechanical signals by reshaping its own microstructure 13, 196. Specifically, under mechanical stimulation (such as cyclic strain 197, fluid flow shear stress 198, oscillatory shear stress 199, vibration 156, specific substrate topography 200, 201, etc.), FAs are formed and FAK is subsequently phosphorylated. This stimulates G-actin to assemble into F-actin, which forms stress fibers together with myosin-2. One end of the stress fibers binds to actin-binding proteins (vinculin and talin) on FAs, and the other connects to the nucleus, thereby conveying signals from FAs to the nucleus 13. During mechanotransduction, myosin-2 acts as a crosslinking agent to harden or soften the actin network by regulating the slip and rearrangement of actin filaments 200, 202. Thus, through regulating myosin-2 activity, many kinases enhance cytoskeletal tension and then participate in mechanosensitive signaling pathways, such as the Rho GTPase protein family: RhoA, Rac1, and cell division control protein 42 homolog (cdc42) 55, 203-206. Conversely, any disruption to myosin-2 hinders the actomyosin from contracting, leading to the alteration of the mechanics inside the nucleus. Subsequently, the activity of osteogenic-related signals, such as ERK and Yes-associated protein (YAP) pathways, is decreased 207. According to this, high levels of actin polymerization and high density of stress fibers is crucial for driving osteogenesis. In addition to acting directly on the nucleus, actin filaments can also transfer mechanical forces to ion channels, such as TRPM7, triggering plasma membrane Ca^2+^ influx 208. It was shown that the disruption of cytoskeletal actin filaments by cytochalasin D (Cyto D), ML-7 or blebbistatin, completely eliminated the force-induced Ca^2+^ oscillations through TRPM7 208, 209. Interstingly, TRPM7-induced Ca^2+^ influx can in turn promoting actin polymerization by increasing intracellular Ca^2+^ concentration 209. This suggests that the interaction between actin microfilaments and TRPM7 during mechanical transduction further enhance the osteogenic effects 209.

In addition to actin, the microtubule dynamics is also proved to be involved in the mechanotransduction pathways underlying MSCs osteogenic differentiation. Although the microtubule cytoskeleton also acts through maintaining the shape of cells and nuclei, it acts passively in the periphery of cells and serves as a “pillar” in the cell structure to support the core cells stably. This is totally different from active stress generated by actin contraction 5, 208. This discrepancy leads to their different ways of altering cell morphology, especially in different microenvironments. In 2D environments, MSCs sense the microenvironment through FAs and the reorganization of actin cytoskeleton, while the microtubule cytoskeleton remains relatively stable 5. The actin cytoskeleton pulls the nucleus on its two separate sides, while the stress fibers push the nucleus downward, flattening the nucleus and allowing MSCs to adjust the morphology freely 210. On the contrast, the 3D environment may limit cell extension, and the overall perceived tension is mainly transmitted through the microtubule cytoskeleton. Microtubule exerts a force opposite to actin, acting on the nucleus. This in turn alters cell and nuclear morphology, accompanied by changes in the heterochromatin in nucleus, thus affecting the gene expression profile of MSC 210. The precise regulation of the microtubule dynamics (polymerization and de polymerization) was confirmed to be important for controlling MSCs fate 211. This is mainly because a complementary force balance is formed between contractile actomyosin filaments and compression-supporting microtubules, supporting the modulation of cell morphorlogy 200. Interestingly, however, microtubule depolymerization, rather than polymerization, appears to favor osteogenic differentiation of MSCs. After microtubule depolymerization, myosin alter its mechanochemical activity by regulating side chain phosphorylation, resulting in an increase in myosin contraction 211. The enhanced contractile force not only directly induces osteogenesis, but also counteracts the traction exerted by the matrix and achieves tensile equilibrium, which in turn further reduces microtubule polymerization and accelerates osteogenesis 5. Moreover, it was confirmed that the passive cytoskeletal support also played an important role in the mechanoactivation of TRPM7 channels and Ca^2+^ influx across the plasma membrane 208.

Compared to actin filaments and microtubules, few studies have been reported on the role of IFs in the osteogenic differentiation of MSCs. A deficient vimentin IF network was shown to decrease the deformability of MSCs, thus affecting osteogenesis 212. Similarly, Stavenschi E et al. demonstrated for the first time that under cyclic hydrostatic pressure (CHP), the remodeling of IFs was required for loading-induced osteogenesis of stem cells 213. To be more specific, under the mechanical pressure, vimentin-based IFs remodel and recoil toward the perinuclear region, inducing downstream osteogenesis 213. These results suggest the potential role of IFs during osteogenesis. Nevertheless, further experiments are required for exploring the specific mechanisms of IFs on loading-induced MSCs osteogenesis.

After sensing mechanical stimulation, the above-mentioned three cytoplasmic cytoskeletons deliver the signal to internal nuclear receptors lamin A/C via Linker of Nucleoskeleton and Cytoskeleton (LINC) complex 214. This complex consists of SUN proteins anchored in the inner nuclear membrane and nesprins anchored in the outer nuclear membrane. In this way , the LINC complex links the cytoplasmic cytoskeleton with the nucleoskeletal lamin A/C. The reorganization of lamins is then achieved in response to mechanical stress 215. Lamin A/C is a kind of intermediate filament proteins, forming a protein meshwork under the nuclear membrane, on which the chromatin is arranged 216. Therefore, mechanical forces lead to alterations in the nuclear envelope structure via lamin A/C mechanotransduction. Subsequently, this structural deformation changes chromatin arrangement and gene expression 215, 216. It was shown that the increased lamin A/C enhaces the stiffness of nucleus, inducing MSCs osteogenic differentiation 214. In contrast, the depletion of lamin A/C contributes to nuclei with irregular shape and severely reduced stiffness 215. Enhanced matrix stiffness 216, 217, hyperboloidal topography 218, convex substrates 219 are demonstrated to improve laminA/C level as well as cell stiffness. For example, lamin A/C levels were 2.5 times higher on convex substrates compared to concave surfaces, and 1.4 times higher compared to flat surfaces 219. Furthermore, the upreguulated lamin A/C may interact with histone deacetylases (HDACs), affecting osteognic gene expression via epigenetic alterations 216. When MSCs were cultured in rigid hydrogel microenvironments, HDAC activity decreased significantly with increased lamin A/C expression, which initiated RUNX2 transcription and promoted osteogenesis 216. Conversely, the disruption of nuclear mechanosensing up-regulated HDACs, preventing epigenetic response as well as osteogenic fate determination. Therefore, lamin A/C plays a determining role during stem cell differentiation.

In short, as the major components of MSCs, the cytoskeleton works together to maintain normal cell morphology and regulate cellular response to mechanical stimuli. Additionally, some specialized structures produced by the cytoskeleton, such as primary cilia composed of microtubules, have been widely recognized as major mechanoreceptors in MSCs 202. Future studies should be dedicated to exploring more on IFs, as well as the interactions between different types of cytoskeletons.

#### 3.1.3 Primary cilium

As a solitary and unfixed mechanoreceptor 220, primary cilium which extends from the cell membrane exist in various tissues, including bone, cartilage, and cardiovascular tissues, etc 202. It consists of nine concentric microtubule filaments, which form the core of the cilium, also known as axoneme 202, 221. Primary cilium senses the mechanical environment through this unique structure and transmits extracellular mechanical signals 202. Recent studies have shown that primary cilium has a considerable role on MSCs after sensing biomaterial-induced physicomechanical stimuli (such as topography 222, 223, fluid shear stress 224, cyclic tensile strain 221, etc.) during driving osteogenesis.

Hoey DA and his colleagues demonstrated for the first time that the primary cilia of stem cells were essential for mechanically-mediated osteogenesis. They stimulated MSCs with OFF in vitro to simulate FSS in physiological environments. The results showed that OFF promoted the proliferation of MSCs and upregulated the expression of osteogenic genes, which was proven to be mediated by primary cilium. On the contrary, hMSCs without primary cilium significantly inhibited osteogenesis in response to mechanical stimulation 224. Similar results were obtained in the study of Chen JC et al 225. Several studies have shown that this mechanically-mediated osteogenic effect is related to the length of cilium 221-223. McMurray RJ et al. found that on the grooved topography, MSCs extended into an elongated morphology toward the groove, and had primary cilia with lengths greater than 3 μm. Nonetheless, such long primary cilia decreased the expression of osteogenic factors 223. Bodle J et al. observed a similar trend. The cells cultured on a hard substrate showed reduced cilia lengths but with stronger osteogenic differentiation ability, compared to those on the softer silicone membrane substrates 221. Additionally, a decreasing trend in the length of cilium was observed as well under cyclic tensile strain 221. Apparently, primary cilium is mechanically sensitive. There are currently two theories to explain this change in cilia length. On one hand, an extended cilium is more likely to detect smaller magnitude changes in the surroundings. On the contrary, the cilium no longer needs such a large “lever arm” to sense mechanical signals when there are larger magnitude mechanical stimuli 221. On the other hand, after exposed to mechanical stimulation, stem cells alter cilia length by reducing actins to regulate downstream osteogenic signaling pathways 223, such as Hedgehog signaling 221, TGF-β signaling 222, Wnt signaling 223, etc.

Additionally, TRPV4 ion channels 226, 227 and G protein coupled receptors (GPCR) 228 are widespread on the primary cilium to mediate mechanical signal transduction and osteogenesis of MSCs. TRPV4 upregulates early osteogenic gene expression through mediating calcium signaling induced by oscillating fluid shear 227. Gpr161, a mechanoreactive GPCR localized in cilia, modulates cAMP and MSC osteogenesis by activating adenylyl cyclase 6 (AC6) 220, 228. Nevertheless, the present study cannot fully explain the specific mechanism of primary cilia promoting osteogenesis in stem cells after sensing biomaterial-induced physical mechanical stimulation, which needs to be further elucidated in further studies.

#### 3.1.4 Ion channels

Recent studies have found that under the mechanical stimulation (such as HP 229, FSS 230, vibration 156, stiffness 230, etc.), MSCs show a rapid increase in the intracellular Ca^2+^ concentration, which drives osteogenesis. This is attributed to the activation of mechanically sensitive Ca^2+^ channels on MSCs, such as Piezo and transient receptor potential (TRP) ion channels.

In 2010, the discovery of Piezo opened a new era of researches on mechanotransduction 231. As one of the most widely studied mechanosensitive cation channels to date, Piezo proteins (Piezo 1 and Piezo 2) are universally localized on the plasma membrane, particularly the lamellipodia and flopodial tips 229. In the presence of membrane tension, Piezo can be activated directly without any additional components 232. The refined structure is the principle reason of its sensitivity to mechanical stimulation. Piezo has a homotrimer structure, similar to a three-bladed propeller, consisting of the peripheral mechanotransduction module and the central ion conduction pore module 233, 234. There are several hypotheses upon the gating mode of Piezo. The force-from-lipids hypothesis states that tension affects lipid-protein interactions between the membrane and ion channel. The protein conformation is subsequently altered, directly activating the channel 235. According to the force-from-filaments hypothesis, interacting cytoskeletal components or ECM is the major cause of the change on Piezo conformation 236. Geng J et al. presented a plug-and-latch hypothesis. More precisely, a plug and a latch exist on each monomer of Piezo. The plug is removed to open ion channels under the pulling of the latch 237.

HP was demonstrated for the first time to transmit mechanical signals via Piezo 229. The Piezo inhibitor GsMTx4 inhibited osteogenic differentiation induced by 0.01 MPa HP loading. Conversely, the Piezo activator Yoda1 drove osteogenesis by upregulating BMP-2, thus enhancing MSC osteogenic differentiation 229. Besides HP, FSS is also involved in signaling through Piezo channels 230. To be more specific, Piezo channels relayed FSS signals to activate Ca^2+^ influx to stimulate Calcineurin. The nuclear factor of activated T cells c1 (NFATc1), YAP1 and β-catenin transcription factors were further activated, to form NFAT/YAP1/β-catenin complex which enhanced osteogenesis. Furthermore, when plated on stiff (40 kPa) hydrogels, MSCs spread to much larger areas with strong nuclear Yap1 localization 230. This suggests that Piezo can sense mechnical stimulus brought by stiffness as well.

TRP is another ubiquitous mechanosensitive channel, consisting of intact membrane proteins with permeable Ca^2+^ 202. The mechanical force is transformed to the channel by surface tension or bending of the lipid bilayer, which leads to a hydrophobic mismatch that opens the channel 238. TRP channels are grouped into seven major subfamilies in mammals according to the nucleotide sequence homology: TRPV (vanilloid), TRPM (melastatin), TRPC (canonical), TRPA (ankyrin), TRPP (polycystin), TRPML (mucolipin), and TRPN (Drosophila NOMPC) 239. Several TRP channels, including TRPV1 156, TRPV4 226, 227, TRPM7 208, 240, 241, have been found to be involved in the mechanical signal transduction of stem cells.

TRPV4 is an extensively investigated TRP channel located in the high strain region, especially in the basal bodies of primary cilia 227. It mainly induces the intracellular Ca^2+^ influx under the stimulus of FSS, as well as the subsequent upregulation of osteogenic genes 226, 227. This is mainly because TRPV4 channels mediate FSS-induced NFATc1 nuclear translocation. Then, NFATc1 and osterix (Osx) form complex to induce the transcription of osteogenic genes of MSCs 226. Moreover, a unique reciprocal feedback loop exists in MSCs between TRPV4 and cell volume expansion, which results in enhanced osteogenic differentiation 105. Under rapid stress relaxation hydrogels or low osmotic pressure, TRPV4 ion channels on the cell membrane increase as cell volume expands. The overexpressed TRPV4 further accelerated cell volume expansion, promoting actomyosin contraction and actin polymerization, thereby driving MSC osteogenesis. In contrast, cells cultured in slowly relaxed hydrogels and high osmotic pressure were limited in volume, even if the TRPV4 was activated 105.

Different from TRPV4, TRPV1 channels sense mechanical forces via nanovibrational stimulation. The influxed Ca^2+^ through the TRPV1 channels triggered the activation of protein kinase C (PKC) and ERK, leading to the activation of downstream β-catenin. β-catenin then translocated into the nucleus and inducing the transcription of osteogenic genes 156.

Recently, TRPM7 is confirmed as one of key mechanical sensors involved in the osteogenesis of MSCs 240, 241. Under different mechanical stimuli including shear stress 241, stretch 208 and pressure 240, TRPM7 channel is activated, resulting in Ca^2+^ release into the cytoplasm. In contrast, TRPM7 mutation can not only completely block the increase of intracellular Ca^2+^ and the nuclear localization of NFATc1 240, but also reduce actin polymerization 209, which is detrimental to osteogenesis. Interestingly, after activated, TRPM7 channels tend to further amplify Ca^2+^ signaling by triggering endoplasmic reticulum (ER) Ca^2+^ release. The activated TRPM7 then interacted with cytophospholipase C (PLC), produced IP3 by hydrolyzing phosphatidylinositol 4,5-bisphosphate (PIP2). Subsequently, IP3 activates inositol trisphosphate receptor type 2 (IP3R2) on the ER conducting Ca^2+^ release 240.

Although the mechanosensitive channels described above can be activated in response to mechanical forces, it is important to note that different types of ions channels may differ in the optimal intensity and duration of stimuli. For instance, it was confirmed that the high-intensity mechanical loading in chondrocytes was mediated by Piezo channels, while the low one was mediated by TRPV4 242. Similar results were found in osteoblastic cells. TRPV4, rather than Piezo1, was sensitive to shear stress upon induction with fluid flow for 5 seconds 243. Nonetheless, comprhensive and precise comparisons have not been performed between TRP and Piezo channels in MSCs.

#### 3.1.5 Gap junction

In addition to the above-mentioned mechanosensors, MSCs communicate with neighbouring cells via gap junctions (GJs) formed by connexins 244, 245. Six identical or different connexins constitute connexons, which exists in pairs to form GJs between adjacent cells as material exchange channels 245. Among various kinds of connexins, Cx43 is the most highly expressed subtype 246. As a communication hub, it functions through special C-termini, helping cells to sense and respond to mechanical stimuli from ECM 245.

Currently, only several kinds of mechanical stimuli have been demonstrated to drive osteogenesis by activating Cx43. Shear stress (0.5 Pa) was confirmed to enhance the osteogenic differentiation of stem cells through Cx43 and Erk1/2 signaling 247. The highest expression of Cx43 and the greatest Erk1/2 activation was shown in the shear stress loading group compared to that in the static group 247. In another study, micro/nano structure was found to promote osteogenic differentiation, related to the upregulated Cx43. To be more specific, on one hand, activated integrins interact directly with Cx43 and induce the opening of Cx43 semi-channels, thus activating cell-cell communication. On the other hand, the activated Cx43 regulates the BMP-2 signaling pathway by partially upregulating BMPR1 on the nanostructure. The overexpressed BMP-2 in turn further regulates Cx43-related intercellular communication 127. Furthermore, micro/nano hybrid structures exhibited a higher stimulative effect on Cx43 expression than micropatterns or nanorods alone. This suggests that micro- and nano-topography play different roles in activating osteogenic signaling pathways. Compared with the micropatterns, nanostructures are more likely to induce stronger Cx43-mediated cell-cell interactions by upregulating BMP-2 expression, resulting in better osteogenesis 127. Although the intercellular communication mediated by Cx43 channels has been demonstrated to play a central role in the osteogenic differentiation of MSCs 244, relatively little is known about Cx43 compared with other mechanosensors. More attention should be devoted to explore other types of mechanical stimuli that act on Cx43, the interaction between Cx43 and other mechanosensors (such as FAs), as well as the Cx43-mediated downstream signaling pathways during osteogenic differentiation 248.

### 3.2 Mechanotransduction pathways

**Figure 3:**
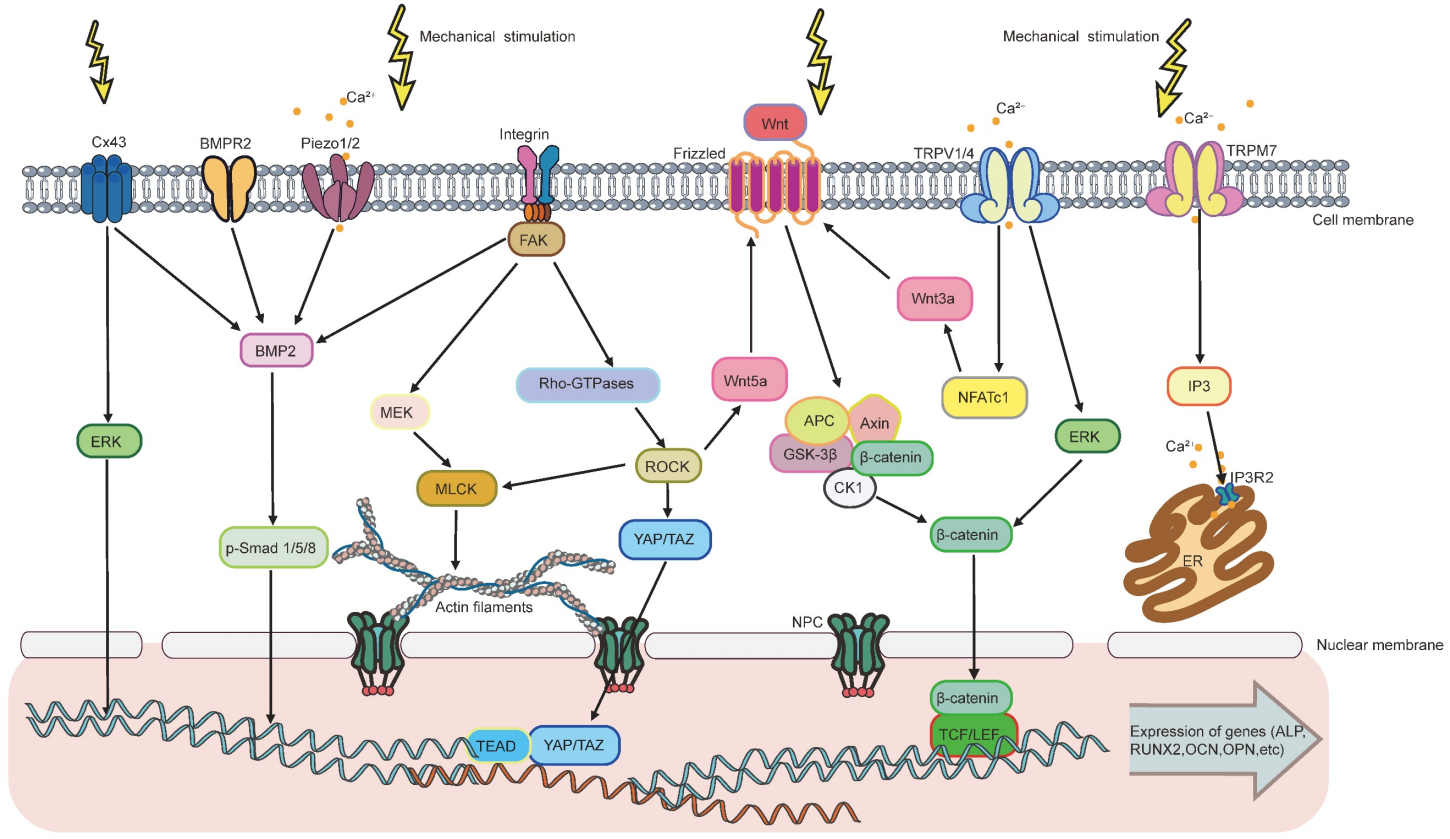
Mechanotransduction pathway involved in biomaterials-induced osteogenic differentiation of MSCs. After integrin and FAs sense the mechanical stimulation, the conformation of FAK changes, exposing phosphorylation sites and activating intracellular osteogenic-related pathways. The activated FAK transmits mechanical signals to the downstream target Rho GTPase, leading to the stimulation of the myosin light chain kinase (MLCK), cooperating with actomyosin and actin filaments to generate the appropriate cytoskeleton tension, which facilitates the entry of YAP into the nucleus by inhibiting LATS1. The canonical Wnt pathway also plays a crucial role. It is activated after Wnt binding and complexing with Lrp5/6 and Fzd, causing Dvl. This destabilizes of the Axin-Apc complex, which facilitates phosphorylation of β-catenin by GSK-3β and Ck1. The unphosphorylated β-catenin then escapes from degradation, allowing it to accumulate in the cytoplasm and translocate to the nucleus. Subsequently, β-catenin initiates the osteogenic gene transcription, acting as a coactivator of with TCF/LEF family. Rho/ROCK is a potential upstream signaling of Wnt/β-catenin sign in stem cells. RhoA and cdc42 inhibit the activity of GSK-3β through phosphorylation, which in turn prevents β-catenin from being degraded. The feedback-regulation is formed between ROCK and Wnt5a. This not only enhances β-catenin transcriptional activity, but also upregulates Wnt signals. Subsequently, Wnt signals in turn improves the activity of ROCK to form a feedback loop. TRP channels are regarded as essential Ca^2+^ channels. TRPV channels sense mechanical forces and permit Ca^2+^ influx, resulting in the activation of downstream β-catenin. TRPM7 channels further amplify Ca^2+^ signaling by triggering ER Ca^2+^ release. The activated TRPM7 then interacted with PLC, produced IP3 by hydrolyzing PIP2. Subsequently, IP3 activates IP3R2 on the ER conducting Ca^2+^ release. MAPK signaling is the downstream of other osteogenic pathways. ERK or p38/MAPK signaling can be activated by FAK, Cx43, Rho-GTPases, etc.

As discussed in the previous section, after mediated by mechanoreceptor, MSCs differentiate into osteogenic lineage through multiple pathways. Several mechanotransduction-associated pathways recently reported in MSCs will be discussed in this section, including YAP/TAZ signaling, MAPK signaling, Rho-GTPases signaling, Wnt/β-catenin signaling, TGFβ superfamily signaling, Notch signaling, etc. **(Figure 3)**.

#### 3.2.1 YAP/TAZ signaling

The Hippo transcriptional coactivator YAP and TAZ are identified as a mechanical rheostat of MSCs, mediating osteogenic differentiation 177. They are highly mechanosensitive to complex microenvironmental cues, resulting in rapid on-off mechanotransduction. When no stimulus is present, large tumor suppressor kinase (LATS)1/2 phosphorylates YAP/TAZ, which leads to cytoplasmic sequestration 177. Conversely, mechanical stress triggers the activated YAP/TAZ to form a complex with Scalloped (Sd) to permit nuclear translocation. YAP/TAZ subsequently interacts with DNA-binding partner TEA domain family member (TEAD) to induce the expression of genes involved in osteogenic differentiation. YAP/TAZ nuclear translocation is driven by FAs formation and the subsequent activation of Rho-GTPase, which promotes actin polymerization and enhanced actin cytoskeleton tension 246, 249. Then, nuclear pores enlarge and YAP translocation occurs due to the cytoskeletal remodeling 14, 249.

Matrix with higher stiffness is confirmed to be an initiator of YAP/TAZ signaling, for it can promote nuclear colocalization of YAP and RUNX2 by progressively organized actin filaments 29, 86, 184, 217. Recently, it was further demonstrated that stiff substrates increased the expression of migration inhibitory factor (MIF) in MSCs, which in turn regulates AKT/YAP signaling to direct osteogenic differentiation 83. Besides actin filaments, biochemical ligand density plays a critical role in stiffness-induced YAP nuclear translocation 187. It was found that YAP translocation was dominated by stiffness only at intermediate ligand densities. However, the low or high ligand densities, rather than stiffness, dominates YAP location 207. Different from the simple 2D culture, YAP/TAZ nuclear localization is less correlated with substrate stiffness in 3D environments 22. Scott KE et al. constructed a 3D spatial model of YAP/ TAZ nuclear translocation in response to stiffness. The aim is to clarify the transfer functions that govern this mechanotransduction pathway 88. It was found that when YAP/TAZ integrates signals from the cytoskeletons, upstream components responded to stiffness changes while dimensionality changes were sensed downstream 88. These findings show the dynamic and complex processes when cells sense their mechanical environment 93.

Topography (such as specific micro-/nano-topography 201, 250, increased surface roughness 14, biomimetic multiscale hierarchical structure 132, curvature 37, 251, etc.) drives osteogenesis through YAP/TAZ activation to a large extent as well. It was demonstrated that integrin clustering and FAs formation were closely related to YAP/TAZ signaling. Larger cell adhesion areas facilitated the activation of YAP/TAZ and subsequent bone formation 250.

#### 3.2.2 MAPK signaling

MAPK signaling pathway consists of ERK, p38 and Jun aminoterminal kinases (JNK) 111. Mechanical cues guide osteogenic differentiation in MSCs mainly through ERK/MAPK signaling 111, 119, 247. For example, ERK1/2 was upregulated after the activation of FAK when mechanical strain was applied on the TiO_2_ nanotubes substrate 252. Shear stress enhanced ERK1/2 phosphorylation, via regulating cell surface channels Cx43 247. Acting as the downstream signal of ERK, CREB is also found to play a critical role when MSCs sense TiO_2_ nanotopography 119. The active ERK1/2 phosphorylates RUNX2, leading to an increase in RUNX2 binding to cofactors CREB, which upregulates the expression of target osteogenic genes 247. Moreover, ERK1/2 and AKT phosphorylation signaling axis was found to be associated with PFKP-mediated-glycolysis, which directly regulated osteogenic differentiation 84. When responding to stiffness cues, PCK2 enhances the rate-limiting metabolic enzyme pallet isoform phosphofructokinase (PFKP), which further activates AKT/ERK1/2 cascades and initiates osteogenesis 84. p38/MAPK signaling can also regulates MSCs fate and activity 131. A recent study revealed that p38 signaling, rather than ERK or JNK, was involved in the chirality-sensing of fate commitment 131. Similar to ERK, p38 interacts with several mechanotransduction-associated signaling pathways as well. Phosphorylated-p38 together with its upstream TRPM7 were both upregulated under shear stress 247. Moreover, it was further indicated in recent studies that ERK1/2 and p38 could both be regulated by Rac1 205.

#### 3.2.3 Rho-GTPases signaling

The Rho GTPase family belongs to the small G protein superfamily 253, which is critical for cell shape remodeling and cytoskeleton organization in response to mechanotranduction 204. Mammalian genomes encode over 20 Rho family members, including RhoA, Rac1, cdc42, etc 253. RhoA and its effector proteins ROCK are most widely studied for their roles in shifting the differentiation potential from adipogenic to osteogenic lineage 254. The enhanced activity of RhoA stimulates the commitment of the osteoblast lineage. Conversely, the reduced activity promotes adipogenic commitment 254. Matrix with high stiffness 85, 255, wavy microstructures 206, fluid flow 148 and nanovibration 156, 256, have been proved to promote osteogenesis of MSCs via the RhoA/ROCK pathway. After sensing mechanical forces, the upregulation of ROCK is observed 148, together with cellular morphological changes due to the increased adhesion-driven cytoskeletal tension 156, 256. Then, osteogenic markers are significantly upregulated and RUNX2 is localized in the nuclei 148. However, the inhibition of ROCK contributes to the disruption of cytoskeleton tension, with downregulated expression of mechanomarkers 133, 257.

The Rho GTPase family are considered as essential regulators of cytoskeleton formation, mainly because their critical roles in FAs-induced osteogenic-related pathways 253. To be more specific, RhoA/ROCK acts as effectors of FAK signaling 253, especially when sensing the roughness and stiffness on matrix 14. After regulated by GTP exchange factors or GTPase activating proteins (GAPs), RhoA is activated. This leads to the stimulation of MLCK, cooperating with actomyosin and actin filaments to generate the appropriate cytoskeleton tension 258. Subsequently, activated RhoA transmits mechanical signals to the downstream target YAP 203, facilitating the entry of YAP into the nucleus by inhibiting LATS1 253. Moreover, it can also promote the phosphorylation of ERK 156, nuclear accumulation of β-catenin 85, 124, thereby activating RUNX2 to enhance osteogenesis. Except for RhoA, Rac1 and cdc42 also play a crucial role in cell differentiation regulation. Rac1 mediates the mechanosensing-dependent osteogenic differentiation of MSCs through regulating downstream ERK1/2 and p38 in MAPK pathway 205, while cdc42 regulates β-catenin signaling activity by phosphorylating GSK-3β 204. This suggests that multiple points of crosstalk are likely to exist between Rho GTPase family and other signals.

Although numerous studies have demonstrated that RhoA/ROCK pathway drives osteogenesis, it is not the central driver compared to other signaling pathways 157. Orapiriyakul W et al. found that reducing intracellular tension via ROCK inhibition lead to only a subtle loss of osteogenesis, which has no significant effect on the overall bone formation 157. It was shown in another study that topography-induced differentiation did not strictly rely on the activation of RhoA. Cells on smooth surface exhibit increased sensitivity to activated RhoA, while those attach to micro/nanostructured surface relies less on it 259. This suggests that other signal molecules might play a dominant role in participating in mechanosensitive regulation. Therefore, much more experimental and theorectical work needs to be done to explore the crosstalk between diverse signaling pathways.

#### 3.2.4 Wnt/β-catenin signaling

The Wnt pathway plays a critical role in MSCs osteogenic differentiation initiated by mechanical forces. The canonical Wnt pathway is activated after Wnt binding and complexing with Lrp5/6 and Frizzled (Fzd), causing Dishevelled (Dvl) 249. This destabilizes of the Axin-Apc complex, which facilitates phosphorylation of β-catenin by GSK-3β and Ck1 260. The unphosphorylated β-catenin then escape from degradation, allowing it to accumulate in the cytoplasm and translocate to the nucleus 260. Subsequently, β-catenin initiates the osteogenic gene transcription, acting as a coactivator of with the transcription factor/lymphoid enhancer-binding factor (TCF/LEF) family 249.

Accumulated experimental studies have demonstrated the involvement of canonical Wnt pathways under mechanical stress. When MSCs sense mechanical stress (such as FSS 230, oscillatory shear stress (OS) 199, low-magnitude and high-frequency (LMHF) vibration 261, etc) or they are seeded on substrates with high stiffness 35, 85, 184, 262, 263 and specific micro/nanotopographies 121, 125, 204, 264, 265, Wnt/β-catenin signaling is initiated and osteogenesis differentiation occurs. Many critical regulators are confirmed to participate in mechanical-stress induced Wnt/β-catenin signaling in MSCs. Piezo1/2 upregulates Wnt/β-catenin and Yap1 activity, by activating Ca^2+^ influx to induce the formation of NFAT/YAP1/β-catenin complex 230. TRPV channels induce the activation of protein kinase C (PKC) and ERK 156, or the nuclear translocation of NFATc1 266, to mediate Wnt/β-catenin activity in MSCs. Rspo1 and its receptor of leucine-rich repeat containing G-protein-coupled receptor 4 (Lgr4) is another novel molecular signal in the upstream of Wnt/β-catenin signaling when transmitting mechanical stimuli to biological signal 267. Rho/ROCK is also a potential upstream signaling of Wnt/β-catenin sign in stem cells 85. Stiff matrices upregulate RhoA, followed by the activation of the Wnt/β-catenin and the promotion of osteogenic differentiation 85. Similarly, cdc42, another member of Rho GTPases family, inhibits the activity of GSK-3β through phosphorylation, which in turn prevents β-catenin from being degraded 204. After β-catenin accumulating in cytoplasm, FAK, paxilin, vinculin, integrin linked kinase (ILK) in FAs can also interact with β-catenin, triggering intracellular β-catenin signaling and promoting its nuclear translocation especially on the stiff substrate 35, 263, 268. It is further shown that a feedback-regulation is formed between ROCK and Wnt5a. Specificially, after sensing the surface structure of the materials, ROCK-signaling pathway is activated, which not only enhances β-catenin transcriptional activity, but also upregulates Wnt5a. Subsequently, Wnt5a in turn improves the activity of ROCK to form a feedback loop 124.

Although the canonical Wnt pathways have been confirmed to exert osteogenic inductive effects under mechanical stress, there still remains some controversy. A recent study found that shear stress induced MSCs to sustain self-renewal capability, rather than differentiatie into osteocytes through inhibiting β-catenin/Wnt signaling and enhancing the expression of SOX2 199. Similar results were obtained in another study 137. This discrepancy could be due to the difficulty in simulating shear stress signals in in vivo microenvironment 199. Therefore, in vivo studies are required to mimic the physiological situation more closely on how mechanical signals alter cell fate choices and cell differentiation of MSCs. In addition, β-catenin was found to act as a negative regulator of osteogenic response. When β-catenin is knocked out, isolated canonical Wnt inhibition increased osteogenic differentiation 262. This is mainly because β-catenin knockdown increases p-Smad1/5, RUNX2, and BMP-4 expression, especially on stiif matrix. Thus, β-catenin may play diverse roles (osteogenic or anti-osteogenic effects) depending on the cell types, species and microenvironment 262.

Except for the canonical Wnt pathway, non-canonical Wnt pathway is also critical for mechanically-induced differentiation 269. It functions independently of β-catenin via two major pathways, the PCP (planar cell polarity) and calcium pathways 262. In the PCP pathway, Fzd activates Rho, Rac, and Cdc42 after associated with Dvl and disheveled-associated activator of morphogenesis (Daam). In the calcium pathway, Ca^2+^ influx occurs through receptor coupled G proteins and phospholipase C 249. Arnsdorf et al. investigated the role of non-canonical Wnt pathway in mechanical forces-induced MSCs for the first time. Exposure to OFF led to translocation of β-catenin and upregulation of Wnt5a. Nonetheless, inhibiting Wnt5a had no significant impact on β-catenin translocation, suggesting the upregulated Wnt5a might be involved in mediating non-canonical Wnt pathway, instead of the canonical one 270. Different from the fluid flow stress, the micro/nano-textured topography down-regulates the ligands of the non-canonical Wnt pathway, including Wnt4, Wnt5a, and Wnt7a 204. Additional research should explore the underlying mechanisms on how various mechanical stress regulates non-canonical Wnt pathway in the future.

#### 3.2.5 TGFβ superfamily signaling

It has been well documented that TGFβ superfamily signaling is crucial for MSCs osteogenic differentiation. As the cytokines of the TGFβ superfamily, BMPs and TGFβ interact with receptors on membranes, then Smad1/5/8 and Smad2/3 are phosphorylated respectively, which are regarded as BMP signaling and TGFβ signaling 222.

BMPs have been extensively studied in recent years 271, they function through interacting with hetero-tetrameric complexes consisting of two dimers, leading to phosphorylation of Smad1/5/8, and the activation of downstream osteogenic-related signaling 271. Nanostructured surfaces have been recently proved to favor cell adhesion and osteogenic differentiation of MSCs, mainly attributed to the activation of BMP/Smad signaling pathway 272. This is because different from other adsorbed proteins that may be affected by the nano-topography, the amount and conformation of BMP-2 remains stable, which results in the excellent osteoinductivity 273. Furthermore, BMP-2 signaling is demonstrated to affect gap junction-mediated intercellular communication in response to micro- or nano-structure of biomaterials 127. To be more specific, osteogenic differentiation induced by surface topography activates intercellular communication, which regulates BMP-2 signaling. Meanwhile, BMP-2 can in turn modulate Cx43-related communication, further driving osteogenesis 127.

BMP signaling also functions in mechano-regulation and stem cell differentiation mediated by stiffness, although Rho GTPase and Wnt signaling may be the ones to play a dominant role 184. Interestingly, when Rho GTPase or F-Actin polymerization is inhibited, a compensatory overexpression in p-Smad1/5 and BMP-2 is observed, instead of the active β-catenin 184. It is further confirmed that BMP-2 might function via a PINCH-1-SMAD specific E3 ubiquitin protein ligase 1 (Smurf1) signaling axis. Generally, Smurf1 binds BMPR2 and controls its degradation in stem cells in response to mechanical signals. After sensing the stiffness in ECM, PINCH-1 is activated, binding directly to the Smurf1 C2 domain where BMPR2 binds. This leads to the inhibition of Smurf1-BMPR2 interaction as well as the degradation of BMPR2, resulting in the consequently augmented BMP signaling and osteogenic differentiation 271.

Different from BMP signaling, TGFβ signaling are poorly investigated. Although it was revealed that TGFβ signaling could be modulated by substrate stiffness and cytoskeletal tension, the underlying mechanisms remain elusive. A recent study showed that surface topography could initiate TGFβ signaling by regulating primary cilia length and TGFβ receptor localization in the cilium 222. However, more exploration is still required on associated pathways in the future.

#### 3.2.6 Notch signaling

Notch signaling has been recently explored as a mechano-transduction pathway in MSCs that regulates cell fate determination and differentiation 274. Among the four Notch receptors (Notch1-4) and five ligands (Dll1, Dll3, Dll4, Jag1, and Jag2), Notch receptor (Notch1, Notch2) and Notch ligand (Dll4, Jag1) serve a dominant function in osteogenic differentiation, especially when MSCs are exposed to low fluid shear stress 275, specific nanostructures 276, cyclic stretching 274, etc. For instance, it was found that low FSS upregulated Dll4 mRNA expression of MSCs, indicating the involvement of Notch signaling in mechanoregulated osteogenic differentiation 275. Notch1, Notch2 genes and their ligand Jag1 was commonly increased through mechanical strain, accompanied by the up-regulated mRNA expression of HES1, HEY1, HEY2, and HEYL, which are the crucial Notch pathway genes 274. Moreover, the active NOTCH signaling has been recently demonstrated to link with the organization of the actin cytoskeleton, but the precise signal still remains elusive 274. Further studies will be required to elucidate the mechanisms underlying the mechanosensitive role of Notch signaling in MSC osteogenic differentiation.

## 4. Perspectives on current understanding

Both physicomechanical properties of biomaterials and mechanical stimulation from external environment play an important role in bone regeneration. MSCs sense specific mechanical signals via mechanosensors on the cell membrane, thereby activating downstream osteogenic-related pathways 3.

Despite intensive research efforts, biomaterials loaded with MSCs have not yet been used in the clinical treatment of bone defects, via mechanical transduction. This is mainly attributed to the degradability of biomaterials after implantation. The physicomechanical properties vary dynamically during degradation 5. Nonetheless, when exploring the optimal mechanical properties such as stiffness and viscoelasticity for osteogenesis in vitro, the degradability of biomaterials and its effects are often ignored. Whether the mechanical properties of materials remain stable during degradation has long been of interest, but less attention has been placed on the effect of mechanical properties on osteogenesis. Another reason that limits mechanical forces to guide the fate of MSCs in clinical practice is that most in vitro studies have been conducted using 2D substrates. On the contrary, the 3D environment in vivo brings different results 277. It is well known that 3D culture is necessary for constructing tissue engineered bone. Compared with 2D culture systems, 3D biomaterials exhibit different cell morphology, cytoskeletal dynamics, and fate determination 5. Therefore, the mechanism of MSCs response to mechanical signals in 3D environment may be the focus of future research.

As traditional static culture cannot provide adequate nutrition and oxygen supply to cells located in the center of the scaffold, the probability of osteolysis is greatly increased 2. Therefore, dynamic culture method becomes the key to solve this issue 140. However, dynamic cell culture through mechanical stimulation remains elusive for subjects in clinical trials. Although mechanical therapies have been moved into the clinic, including low-level vibrations, dynamic hydraulic stimulation (DHS) and extracorporeal shockwave therapy (ESWT), however, the efficiency of these mechanical therapies in bone repair remains controversial 278. Therefore, future researches may focus on the development of more efficient mechanical therapies, as well as the combined treatment of mechanical properties of biomaterials and external mechanical stimulation on MSCs.

Although numerous studies have been conducted on promoting osteogenic differentiation of MSCs under mechanical stimulation, the interaction between MSCs and other cell types remain poorly understood, including vascular endothelial cells, osteocytes, osteoblasts, etc 95. In contrast, further researches on macrophages are underway. It was previously reported that mechanical force exerted by orthodontic process induced the targeted activation of Smad1 by macrophages-derived ubiquitin carboxyl-terminal hydrolase isozyme L3 (an exosome) to promote osteogenesis of MSCs 279. Moreover, the polarization of macrophages is of more concern recently. Generally, macrophages are divided into pro-inflammatory M1 phenotypes and anti-inflammatory M2 phenotypes 95, which function at different stages of bone defect healing cascades and regeneration. Several biomaterial characteristics have been shown to guide macrophage polarization, including stiffness 95, topological structure 280, 281 and cytokines 282, 283. He *et al.* demonstrated that macrophages cultured under both 2D and 3D conditions exhibited M2 polarization at low stiffness and M1 polarization at high stiffness by using gelatin materials with adjustable stiffness 95. It was further demonstrated that it was the altered lipid metabolism that led to the 12-lipoxygenase mediated change in macrophage phenotype 94. However, different from the results of culturing MSCs alone 85, when macrophages were also co-cultured on a high-stiffness matrix, the pro-inflammatory phenotype of M1 macrophages impaired the osteogenesis process. Interestingly, the most recent developed materials show totally different results. The decellularized placental sponge with native biological structure can promote M2 macrophages polarization as well as the osteogenesis crosstalk between two cells 284. Another similar material, decellularized cartilage matrix with appropriate IL-4 delivery, is also a good immunomodulatory strategy, which may be related to the regulation of macrophage polarization directed by collagen type Ⅵ 285-287.

Surface topography of biomaterials has also been shown to be one of the key factors affecting macrophage polarization and osseointegration efficiency 280, 288, 289. Altering the roughness, surface modification, etc, can drive cellular migration and polarization, thus down-regulating the initiation of pro-inflammatory cascades 289. Compared with the traditional Ti coating, the micropatterned Ti surface promotes the M2 polarization 280. Furthermore, Wang et al. discovered that the alteration in the diameter of micropatterned nanotubes also affected the polarization direction: macrophages on small diameter(30nm) nanotubes were more inclined to M2 polarization, and those on large diameter(100nm) nanotubes had the opposite results 281. Similar conclusion was obtained by using Ti nanotubes with diameters of 80-100nm 290. And by blocking the secretion of MSCs exosomes, it was concluded that M1 polarization was regulated by the paracrine pathway of MSCs. Moreover, the nanofiber membrane can also serve as a promoting surface design. The membrane with lattice topology prepared by electrospinning method is proved to recruit macrophages 291, and the layered scaffold with nano-morphology fiber membrane combined with mineralized particles can induce M2 polarization 292.

## 5. Conclusion

MSCs-directed osteogenic differentiation by physicomechanical stimulation has become a growing area of research in recent years. By altering the stiffness, viscoelasticity, and topography, or exerting external loading, mechanical signals can influence the development of MSCs via distinct pathways, thereby controlling their fate more precisely. However, additional studies are needed to fully elucidate more detailed mechanotransduction mechanisms, including how mechanoreceptors and ion channels respond to mechanical stimuli, and downstream pathways that regulate osteogenic-related transcription factors. In addition, the development of novel biomaterials that provide more stable and independently regulated properties are still required for better guidance in BTE. In conclusion, it can be predicted that mechanically stimulated MSCs-laden materials will be an ideal source for BTE. This emerging research area warrants further study, which may offer substantial clinical benefits in the near future.

## Figures and Tables

**Table 1:** Effects of cell-laden biomaterials on MSCs osteogenic differentiation induced by matrix stiffness

Stem cell source	Biomaterial	Stiffness range	Functional activities	Ref
hMSCs	Macro-porous recombinant elastin-like protein (ELP) substrates	0.5-50 kPa	Increase adipogenic and osteogenic differentiation markers with increasing stiffness.	4
	PA hydrogels	3, 14, 38 kPa	Stiffness-induced YAP nuclear translocation was only observed when hMSCs were cultured on hydrogels coated with intermediate concentration of fibronectin.	93
	Methacrylate gelatin (GelMA) hydrogels	3.8, 31.3 kPa	Osteogenesis were enhanced on very soft hydrogels with high surface roughness.	14
	Electrospun PLLA ultrafine fibers	77.4, 729,1124 MPa (Young's modulus)	A stiff substrate downregulates the stemness property of hMSCs and directs the cells toward the osteogenic lineage.	83
	3D bioprinted cell-laden scaffolds	0.66, 5.4 kPa	Soft scaffolds had enhanced ALP activity and stimulated osteogenic differentiation than stiff ones.	26
	Methacrylated hyaluronic acid (MeHA) hydrogels	5, 12, 23 kPa	When cells had an optimal volume, cells could form clear stress fibers and FAs on soft, intermediate, or stiff matrix.	22
rat MSCs	3D DBM scaffold	66.06, 26.90, 0.67 MPa (compressive modulus)	Low scaffolds could promote the osteogenic differentiation of MSCs.	91
	GelMA hydrogels	6, 10, 25 kPa	Osteogenic differentiation was increased with the elevation of 3D ECM stiffness.	84
	Magnetic liquid metal (MLM) scaffold	3.58-14.32 MPa	MLM scaffold has good biocompatibility and can promote the osteogenic differentiation of MSCs.	87
	PEG/SF/HAp scaffolds	80.98-190.51 kPa	The scaffolds fabricated with HAp (50 mg) increased cell adhesion and viability as well as the expression of all the osteogenesis-related markers.	89
mouse MSCs	Alginate-gelatin (Alg-Gel) composite hydrogels	50 kPa, 225 kPa (Young's modulus)	Higher expression of adipogenic and osteogenic markers were shown in stiffer 3D-bioprinted matrices.	86
	DBM scaffolds	0.67 MPa	Low matrix stiffness could polarize macrophages into an anti-inflammatory phenotype, and specialized pro-resolving lipid mediators (SPMs) biosynthesis beneficial for the osteogenesis of MSCs.	94
	Transglutaminase cross-linked gelatin (TG-gel)	60.54, 1.58 kPa (yield strength)	Low-stiffness TG-gels promoted BMSC proliferation, whereas high-stiffness TG-gels supported cell osteogenic differentiation.	95

**Table 2:** Effects of cell-laden biomaterials on MSCs osteogenic differentiation induced by viscoelasticity

Stem cell source	Biomaterial	Initial elastic modulus	Half stress relaxation time (τ1/2)	Functional activities	Ref
hMSCs	Alginate hydrogels	-	20 s	Significant increases were observed in calcium deposition by MSC spheroids loaded with BMP-2-HA in viscoelastic gels.	30
		-	14.4±1.0 s	Modulating viscoelastic properties of biomaterials, in conjunction with dual peptide functionalization, can simultaneously enhance multiple aspects of MSC regenerative potential.	97
	Boronate-Based Hydrogels	14.1±2.7 kPa	-	The fast relaxation matrix mechanics are found to promote cell-matrix interactions, leading to spreading and an increase in nuclear volume, and induce yes-associated protein/PDZ binding domain nuclear localization at longer times.	15
	Hyaluronic acid-collagen hydrogels	-	560-2200 s	Faster relaxation in the interpenetrating network hydrogels promotes cell spreading, fiber remodeling, and FA formation.	107
mouse MSCs	RGD-coupled alginate-PEG hydrogels	3 kPa	A few hours to a few minutes	Faster relaxation in RGD-coupled alginate-PEG hydrogels led to increased spreading and proliferation of fibroblasts, and enhanced osteogenic differentiation of MSCs.	28
	RGD coupled alginate hydrogels	17kPa	1 min	Cell spreading, proliferation, and osteogenic differentiation of MSCs are all enhanced in cells cultured in gels with faster relaxation.	27
	Alginate hydrogels	20kPa	-	MSCs in viscoelastic hydrogels exhibit volume expansion during cell spreading, and greater volume expansion is associated with enhanced osteogenesis.	105

**Table 3:** Effects of cell-laden biomaterials on MSCs osteogenic differentiation induced by topography

Cell type	Material	Surface patterns	Result	Ref
hMSCs	PI	Micro-patterns Width: 2-15μm Depth: 2μm	15 μm ridges increased adipogenic differentiation whereas 2 μm ridges enhanced osteogenic differentiation.	9
		Nano-patterns Diameter: 600μm Depth: 200nm Periodicity: 650nm	Nano-patterns increased differentiation towards both osteogenic and adipogenic lineages.	9
	PDTEC PS	Co-continuous ribbons Spacing: 48±5μm Height: 200nm	Co-continuous topographies favor cytoskeletal anisotropy, FA maturation and osteogenic differentiation.	42
	HA	Micro/nano hybrid structure Width: 28μm Space: 24μm Diameter:70-100nm	The micro/nano hybrid structure significantly enhanced the cell behavior including the adhesion, proliferation and osteogenic gene expression.	127
	Quartz	Chiral geometry Linewidth: 2μm Spacing: 2μm Depth: 3μm	Cell adhesion, proliferation, and differentiation are greatly enhanced for cells cultured on dextral geometry than those on sinistral geometry.	131
	Silicone	Periodic nanopillar arrays Diameter: 54-105nm Periodicity:70-201nm Height: 39-85nm	The nanopillar arrays enhance osteogenic differentiation of hMSCs, dependent on the age of the donor.	58
		Multiscale hierarchical topography	The 0.5⊥3∥25 substrate, resembling collagen topography the most, exhibits the highest osteogenesis.	132
	CDMs PDMS	Wave-like structure Amplitude: 0.4, 2.2μm	CDMs and topography synergistically enhances osteogenic differentiation.	116
	PDMS	Wave-like topographies Wavelength: 0.5,3,10,27μm	Compared to W27, W3 showed the enhanced stiffness of stem cell, promoting higher degree of osteogenic differentiation.	37
	TiO_2_ nanotubes	TiO_2_ nanograin with the nanopore surface Width: 50-60nm Diameter: 30-40 nm	The expression of p-ERK and p-CREB increased in the TiO_2_ nanograin with the nanopore surface compared to the micro rough and nanotube surfaces.	119
Rat MSCs	HAp	Micropatterns Height: 11.38±0.58μm Length: 63.87±3.41μm Width: 43.31±2.55μm	The micro-patterned topography and Sr-doping had a synergetic effect on the adhesion, growth and osteogenic differentiation of BMSCs.	111
	BaTiO_3_/ poly-(l-lactic acid) fibrous scaffolds	Randomly oriented electrospun	The topographical structure and electrical activity have combining effects on cell attachment, growth, and osteogenic response.	130
	TiO_2_ nanorod array	Nanoscale geometry Length: 1.5μm Diameter: 100nm	A TiO_2_ nanorod array promotes the osteogenic differentiation of MSCs, while a TiO_2_ ceramic with a smooth surface suppresses it.	59
MSCs	PCL	Micro-grooves Width: 16μm Height: 6μm	The space constraint inhibits the extension of actomyosin cytoskeleton, instead, pseudopodia lead to cell polarization.	126
		Nano-grooves Width: 400nm Height: 500nm	The adhesion induction leads to the formation of FAs, promoting the osteogenic differentiation of stem cells.	126

**Table 4:** Effects of cell-laden biomaterials on MSCs osteogenic differentiation induced by external mechanical stimulation

Loading	Loading Regime	Scaffold	Osteogenesis	Ref
Compression	42%, 0.3Hz, 3h/day for 21 days	GelMA	ALP, RUNX2, OCN, OPN, Mineral deposition (+)	140
	5-10%, 1Hz, 8h/day for 6 days	Collagen	BMP-2 (+) RUNX2, Col-1 (-)	142
	5-20%, 1Hz, 2h/day for 28 days	Poly(ε-caprolactone)	RUNX2 (+) COL1A1 (-)	139
	20-60%, 0.75Hz, 4h/day for 7 days	Octacalcium phosphate-gelatin	OCN, OPN, Col-1 (+)	143
Stretching	10%, 1Hz, 4h/day for 21 days	Fibrin hydrogel	ACAN, SOX9, BMP-2, RUNX2, OPN, COL1A1 (+) ALP (-)	144
Perfusion	3ml/min (0.2 dynes/cm2) for 14 days	HA-PLGA	IBSP (+)	145
	7ml/min for 6 weeks	Alginate and gelatin-based hydrogel	Mineral deposition (+)	146
	1ml/min, 30min/day for 3 weeks	Collagen-HA	OCN, OPN, Collagen, Mineral deposition (+)	147
	0.8ml/min, 8h/day for 21 days	LTMC	Collagen, ALP (+)	148
	6.3 cm^3^ min^-1^ for 0-2 weeks in the standard medium and 0-2 weeks in a differentiation medium	Apatite-Fiber	ALP, Calcification (+)	149
	1.7ml/min, 5min every 15 min/day for 21 days	RCP, MgAp	Cell viability (+)	150
	116μm/s for 21 days	Collagen coated with Mg -doped HA	ALP, OCN, OPN, BMP-2 (+)	151
	3μl/min, 6h /day for 7 days	HA (750-900μm)	ALP (+)	51
	10ml/min for 14 days	Fibrin breads	OPN, RUNX2, VEGF (+)	152
	1.7ml/min for 21 days	Chitsan/HA	Collagen, Osteocalcin, Calcium deposition (+)	153
	2ml/min for 14 days	HA-PCL	ALP, RUNX2 (+)	154
Rotating, perfusion and compression	0.22%, 1Hz, 5rpm /min, 4h/day for 2 weeks	PCL/TCP	RUNX2, COL1A1 (+)	155
Vibration	30nm amplitude, 1000Hz for 21 days	Collagen	RUNX2, Collagen, ALP, OCN, OPN, BMP-2 (+)	156
	90nm amplitude, 1000Hz for 9 days	Collagen	RUNX2, OSX, ALP, OCN, OPN, ON (+)	157
	30nm amplitude, 1000Hz for 3 weeks	Collagen	RUNX2, OSX, OPN, OCN, ALP (+)	138

## Abbreviations

AC6: adenylyl cyclase 6
BTE: Bone tissue engineering
CDM: cell-derived matrices
cdc42: control protein 42 homolog
CHP: cyclic hydrostatic pressure
Cyto D: cytochalasin D
PLC: cytophospholipase C
Daam: disheveled-associated activator of morphogenesis
Dvl: Dishevelled
DHS: dynamic hydraulic stimulation
ERK: extracellular-signal-regulated kinase
ESWT: extracorporeal shockwave therapy
FSS: fluid shear stress
FAs: focal adhesions
Fzd: Frizzled
GPCR: G protein coupled receptors
GJs: gap junctions
GAPs: GTPase activating proteins
HDACs: histone deacetylases
hASCs: human adipose-derived stem cells
hUCS: human umbilical cord serum
HP: hydrostatic pressure
HAp: hydroxyapatite
IP3R2: inositol trisphosphate receptor type 2
IACs: integrin adhesion complexes
ILK: integrin linked kinase
IFs: intermediate filaments
IHP: intermittent hydrostatic pressure
JNK: Jun aminoterminal kinases
LATS: large tumor suppressor kinase
Lgr4: leucine-rich repeat containing G-protein-coupled receptor 4
LINC: Linker of Nucleoskeleton and Cytoskeleton
LMHF: low-magnitude and high-frequency
MSCs: mesenchymal stem cells
MIF: migration inhibitory factor
MNNTs: multiwall carbon nanotubes
nHA: nano-hydroxyapatite
NFATc1: nuclear factor of activated T cells c1
OFF: oscillatory fluid flow
OS: oscillatory shear stress
Osx: osterix
PCP: planar cell polarity
PIP2: phosphatidylinositol 4,5-bisphosphate
PFKP: phosphofructokinase
PDTEC: poly(desaminotyrosyl-tyrosine carbonate)
PA: polyacrylamide
PDMS: polydimethylsilane
PEG: polyethylene glycol
PEG/SF/HAp: polyethylene glycol/silk fibroin/hydroxyapatite
PI: polyimide
PLLA: poly-l-lactic acid
PS: polystyrene
PKC: protein kinase C
Sd: Scalloped
Ta: Tantalum
TEAD: TEA domain family member
3D: three-dimensional
TCF/LEF: transcription factor/lymphoid enhancer-binding factor
TRP: transient receptor potential
YAP: yes associated proteins
